# Innovative Ultrasound-Guided Erector Spinae Plane Nerve Block Model for Training Emergency Medicine Physicians

**DOI:** 10.21980/J8PW7D

**Published:** 2025-04-30

**Authors:** Jose Correa Ibarra, Amelia Crowley, Sydney Hughes Lindros, Kevin B Walker, Caroline Astemborski, Phillip Moschella

**Affiliations:** * Prisma Health Upstate, Department of Emergency Medicine, Greenville, SC; ^ Clemson University, Department of Public Health Sciences, Clemson, SC

## Abstract

**Audience:**

This ultrasound-guided erector spinae plane (ESP) nerve block model is designed to instruct emergency medicine (EM) attending and resident physicians. However, this innovation is appropriate for all levels of learners, including medical students, advanced practice clinicians (APCs), and clinicians in other medical specialties.

**Introduction:**

The ESP nerve block is a relatively new regional anesthesia technique that involves injection of local anesthetic along the fascial plane below the erector spinae muscles.^1^–^3^ The ESP nerve block was first described in 2016 by Forero et al. to help manage severe thoracic neuropathic pain resulting from malunion of multiple rib fractures and metastatic disease of the ribs.^1^ The block has since emerged as a safe, feasible and effective analgesic intervention for various pathologies, including management of pain for acute rib fractures.^2^,^3^ However, barriers to implementation into routine practice in the emergency department (ED) exist due to gaps in knowledge about the block and a lack of training.^4^ We created a novel, inexpensive, and portable ultrasound-guided ESP nerve block model that can be used to facilitate training for EM physicians and residents.

**Educational Objectives:**

This innovation model is designed to facilitate hands-on training of the ultrasound-guided ESP nerve block using a practical, realistic, and cost-effective ballistics gel model. By the end of this training session, learners should be able to: 1) identify relevant sonoanatomy on the created simulation model; 2) demonstrate proper in-plane technique; and 3) successfully replicate the procedure on a different target on the created training model.

**Educational Methods:**

We created a cost-effective ESP nerve block model using a 3-D printed spine and ribcage suspended in ballistics gel that is compatible with ultrasound. The use of ballistics gelatin in the model closely simulates the viscosity and density of animal tissue, allows for ultrasound use, and is cost-efficient and more feasible than other organic models because it can be easily melted and re-used.^5^ At the time of this model’s creation, the only previous approach to creating an ESP model was a porcine model that used meat cuts from the lower thoracic region and spine. However, the major limitation of this porcine model was its limited shelf life.^6^ The created ESP model was incorporated into a hands-on training module that took place one to two times per week over two months. Additional sessions were incorporated on a case-by-case basis. All participants were first given access to an educational ESP Nerve Block PowerPoint presentation to be reviewed prior to attending in-person sessions. The training sessions were promoted through weekly email reminders containing the dates and a link to an online sign-up sheet. Additionally, on training days, our project director actively sought to recruit available participants on-shift. Each training day, a one-to-two-hour window was made available for participants to attend. Each training session was conducted with a small group of four or fewer trainees beginning with a short didactic lecture presented by a lead instructor, either the Associate Research Director of Emergency Medicine or the Medical Director for Division of Pain Medicine, followed by live demonstration of the nerve block using the ESP model. Participants were then given the opportunity to practice on the ESP model. Sessions ended when all participants demonstrated proper and successful technique with the model, reported adequate confidence with the block, and all questions were addressed. Feedback on technique was provided throughout the training session by the lead instructor.

**Research Methods:**

Post-education surveys were distributed to all participants electronically to assess training impact. The survey collected data on the participants’ title, prior experience performing ESP nerve blocks, competency of the teaching model, and their comfort with performing the block after the training. The Institutional Review Board (IRB) reviewed and deemed this project exempt from full board review.

**Results:**

Thirty-four participants attended the in-person training sessions, consisting mainly of EM attending (16/34; 47%) and resident (13/34; 38%) physicians. Fourteen (14/34; 41%) participants returned completed surveys, of which 50% were residents (7/14; 50%) and 50% attending physicians (7/14; 50%). The majority (12/14; 86%) of respondents reported no prior experience in performing an ESP block with only 14% (2/14; 14%) reporting performing fewer than two ESP nerve blocks per year. All respondents (14/14; 100%) agreed or strongly agreed that the education session with the ESP model improved their confidence, knowledge, and skills to perform the block. All (14/14; 100%) agreed or strongly agreed that they felt confident in their ability to use ultrasound to identify landmarks on the model pertinent to performing the ESP block. All (14/14; 100%) reported that they felt that the material presented during these training sessions was relevant to their practice in the ED, within their scope of practice, and part of their job as an ED physician. All (14/14; 100%) reported they felt performing ESP blocks in the ED could positively impact patient outcomes and reported an increased likelihood of performing the ESP block in the ED following this training session. Lastly, respondents were asked to list any barriers that might inhibit them from performing the ESP block on shift, in addition to any strategies to facilitate ESP block use. Four participants (4/14; 29%) reported barriers to performing an ESP block including time constraints (50%) and patient mobility limitations (50%). Twelve participants (12/14; 86%) reported facilitators to performing ESP blocks, the most common of which being easier access to supplies and assistance with procedure setup (43%), followed by increased education sessions (21%).

**Discussion:**

Our survey results indicate that our learners perceived an increase in knowledge, confidence, and skills in performing ultrasound-guided ESP blocks after using our innovative model as a hands-on teaching tool during a training session. A simple 30-minute training session with a novel ballistics gelatin ESP model can improve confidence, knowledge, and skills in performing this block in the ED, even amongst nerve block naive physicians. Additionally, by identifying barriers to the use of the ESP block in the ED, researchers can create strategies to mitigate these challenges to increase utilization of these procedures for appropriate patients in the ED. These strategies include but are not limited to addressing ways to mitigate time constraint issues, patient mobility limitations, access to supplies, assistance with procedure set up, and increasing education sessions to increase physician comfort with successful completion of the procedure.

**Topics:**

Erector spinae plane nerve block, ultrasound, regional anesthesia, rib fractures, ballistics gel model, hands-on training.

## USER GUIDE

**Table:** 

List of Resources:	
Abstract	1
User Guide	4
ESP US Nerve Block PowerPoint	10

**Learner Audience:**

Medical Students, Interns, Junior Residents, Senior Residents, EM Attendings, EM APCs

**Time Required for Implementation:**

Construction of the model after the artificial spine and ribcage have been printed takes approximately five to eight hours (3D printing takes about 22 hours). Training sessions take approximately 30 minutes to complete: 5–10 minutes for introductory didactic lecture, five minutes for instructor demonstration of the ESP nerve block on model, and 15–20 minutes for participants to practice.

**Recommended Number of Learners per Instructor:**

Small groups of less than four participants per instructor or two participants per model.

**Topics:**

Erector spinae plane nerve block, ultrasound, regional anesthesia, rib fractures, ballistics gel model, hands-on training.

**Objectives:**

By the end of the training session with the ESP model, learners should be able to:

1. Identify relevant sonoanatomy on the simulation model including the transverse process and ribs.
2. Demonstrate proper in-plane technique to maintain continuous visualization of the entire needle during procedure.
3. Replicate the procedure on the training model on multiple transverse process or rib targets.

### Linked objectives, methods and results

In the ED, the use of ultrasound-guided regional nerve blocks for acute pain have become an acceptable alternative or adjunct to opioids and other pharmacotherapeutics.^7^ Despite its encouraging safety profile and efficacy, the ESP nerve block is grossly underutilized in the ED mainly due to existing gaps in knowledge about the block and a lack of training.^4^ The development of this ESP nerve block model aims to facilitate hands-on training for EM attending and resident physicians in acquiring the proper skills, knowledge, and confidence needed to perform an ultrasound-guided ESP nerve block.

Learners are familiarized with the block and the relevant anatomy through an introductory didactic presentation and instructor demonstration on the model. Next, learners will first begin by using the ultrasound probe to identify the relevant sonoanatomy, including the transverse process (TP) and ribs. (Objective 1).

Once the target TP or rib is identified, learners will incorporate in-plane ultrasound-guided techniques with the needle to reach the desired target on the model. (Objective 2). An in-plane ultrasound-guidance approach is strongly recommended for this single injection nerve block because it allows for full visualization of the needle at all times, contrary to the out-of-plane approach, which occasionally generates a limited view of the needle and often makes it hard to decipher which part of the needle is being visualized.^8^

Finally, learners are asked to replicate the procedure several times on different TP or rib targets on the model, allowing for repetitive practice to build confidence and skill. (Objective 3).

### Recommended pre-reading for instructor

- Crowley, Amelia. PowerPoint: Erector Spinae Plane Block in the ED. 2024.
- Erector Spinae Plane Nerve Block. NYSORA. https://www.nysora.com/erector-spinae-plane-block/
- Forero M, Adhikary SD, Lopez H, Tsui C, Chin KJ. The erector spinae plane block: A novel analgesic technique in thoracic neuropathic pain. *Reg Anesth Pain Med.* 2016;41(5):621–627.
- doi:10.1097/AAP.0000000000000451
- Luftig J, Mantuani D, Herring AA, Dixon B, Clattenburg E, Nagdev A. Successful emergency pain control for posterior rib fractures with ultrasound-guided erector spinae plane block. *Am J Emerg Med*. 2018;36(8):1391–1396. doi:10.1016/j.ajem.2017.12.060

### Learner responsible content (LRC)

All participants should have fundamental ultrasound knowledge and should be able to identify all pertinent anatomical landmarks. It is also highly recommended for individuals to review the associated PowerPoint content before attending the session, as well as the following web resource:

- Erector Spinae Plane Nerve Block. NYSORA – https://www.nysora.com/erector-spinae-plane-block/

### Implementation Methods

We first began with developing and constructing the ultrasound-guided ESP nerve block model. The ESP model was produced by our Simulation Center at the University of South Carolina School of Medicine, Greenville. A 3D printer was used to produce an artificial bony segment of the spine and ribcage, which was then suspended in melted ballistics gel.

Once created, the model was integrated into in-person educational training sessions designed for small groups (less than four individuals, two per model created). Training was scheduled tentatively one to two times per week over two months. Each training day, an open window of one to two hours was made available by which multiple training sessions could take place. Before the in-person training started, learners had access to an educational PowerPoint presentation lecture tailored to the ESP nerve block for reviewing.

Each individual education session was broken into three parts, beginning with a short didactic lecture, followed by demonstration, and then practicing of the nerve block on the training model. Questions and clarifications were addressed throughout the training session by the instructors.

Hands-on training sessions concluded once all participants were able to practice the block on the simulation model and felt confident in doing so. Post-training surveys were then distributed via QR code and email with responses collected anonymously via RedCap.

### List of items required to replicate this innovation

1. 3D-printed spine segment with associated ribcage
2. Humimic ballistics gelatin – https://humimic.com/product/diy-ultrasound-phantom-gel/
3. 9” × 6” × 2” aluminum baking pan
4. Slow cook burner
5. Ultrasound machine with linear probe
6. Ultrasound gel
7. Echogenic needles

### Approximate cost of items to create this innovation

The cost to create this model was approximately $250. Worth mentioning, the model has a long shelf life and can be melted and reshaped, making it easily reusable and cost-efficient.

### Detailed methods to construct this innovation

The construction of this innovative model consisted of two key components: a 3D-printed artificial bone segment of spine and partial ribcage that was then suspended in melted ballistics gelatin.

1. A publicly available CT scan file of a segment of the spine and ribcage was used to 3D print an artificial bone model on a ProJet MJP 2500 machine.^9^ If a 3D-printer is not available, Protolabs Network by Hubs offers 3D printing services. A quote was obtained for the unmodified CT scan file for approximately $190.00.^10^ Other 3D printing services could be explored for price comparisons (The UPS Store®, Xometry®, RapidMade, Inc, Shapeways, Craftcloud®).[Fig f1-jetem-10-2-i1]
  **Figure 1 f1-jetem-10-2-i1:**
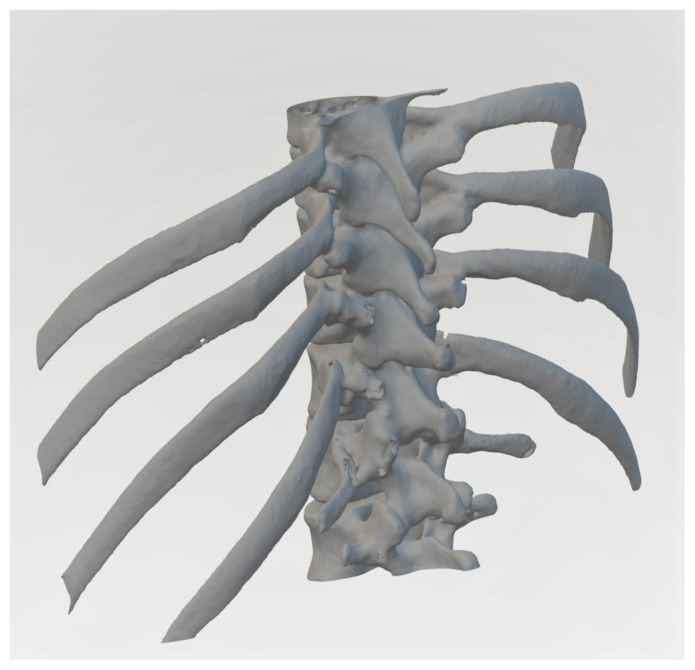
Prevue Medical. Lower Spine and Ribcage from Chest CT by Prevue. In: Thingsverse. https://www.thingiverse.com/thing:54669/files. Published April 7, 2013. Accessed April 15, 2024. CT scan file of the segment of spine and ribcage used to create the model. The file was further modified to reduce the size, which involved cropping of the two inferior vertebrae and shortening of the ribs by approximately half the length.
2. Upon completion of the 3D printing process (approximately 22 hours), the model was then placed into a hot water bath to remove the external support material. The model was then additionally washed with soap and water to remove any remaining debris.[Fig f2-jetem-10-2-i1]
  **Figure 2 f2-jetem-10-2-i1:**
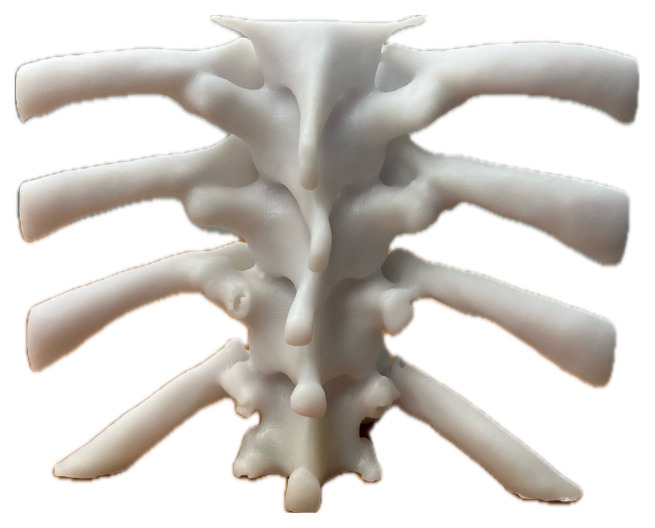
Author’s own image. Finalized 3D-printed spine and ribcage model prior to suspension in ballistics gel.
3. Humimic® ballistics gel was melted down on a hot plate at low-to-medium heat until fully liquefied.
4. The 3D-printed artificial bone model was placed into a 9” × 6” × 2” aluminum baking pan with the spinous processes facing down.[Fig f3-jetem-10-2-i1]
  **Figure 3 f3-jetem-10-2-i1:**
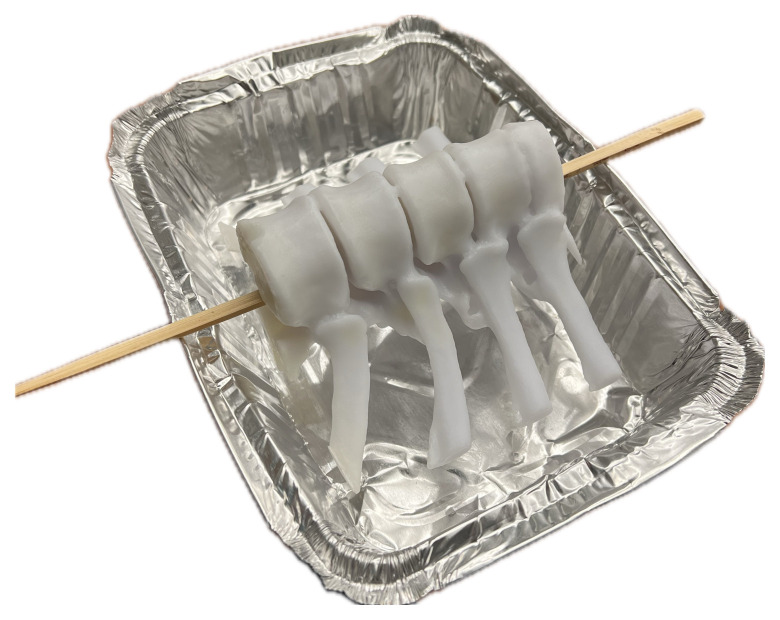
Author’s own image. When setting up the 3D printed model, a skewer was inserted through the vertebral foramen. This allows for the ribs to be suspended at a realistic depth when covered by the ballistics gel.
5. Liquefied ballistics gel was then added slowly, covering most of the 3D-printed artificial bone model.[Fig f4-jetem-10-2-i1]
  **Figure 4 f4-jetem-10-2-i1:**
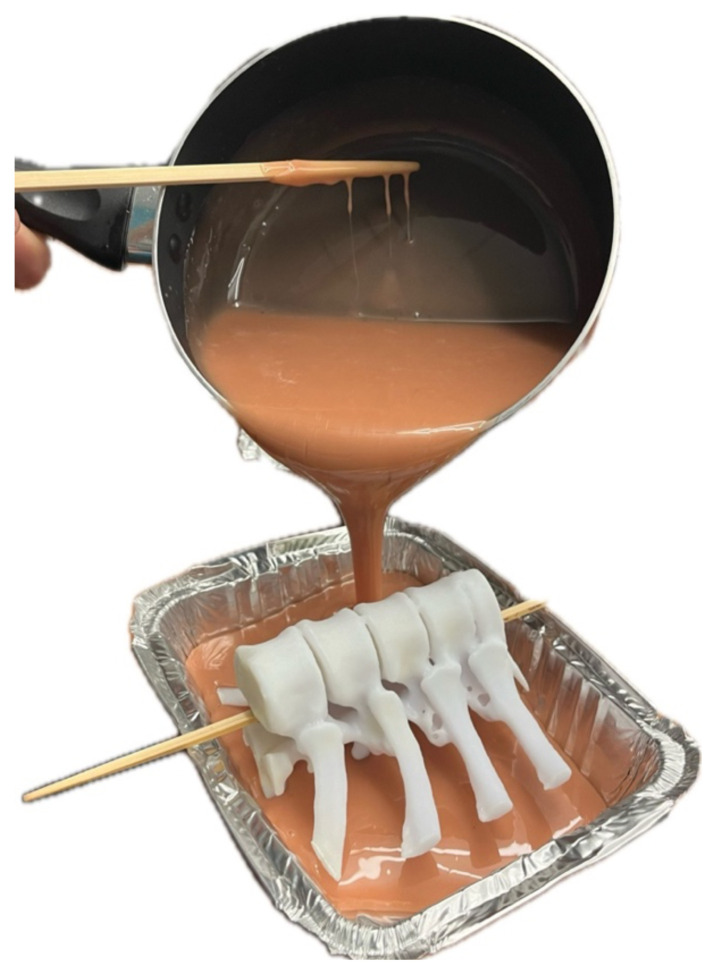
Author’s own image. Enough ballistics gel was added, engulfing the ribs completely and leaving the vertebral bodies exposed.
6. The ballistics gel was allowed to settle completely (approximately 5–6 hours). Once fully solidified, the model should detach easily from the aluminum pan and maintain shape integrity.[Fig f5-jetem-10-2-i1]
  **Figure 5 f5-jetem-10-2-i1:**
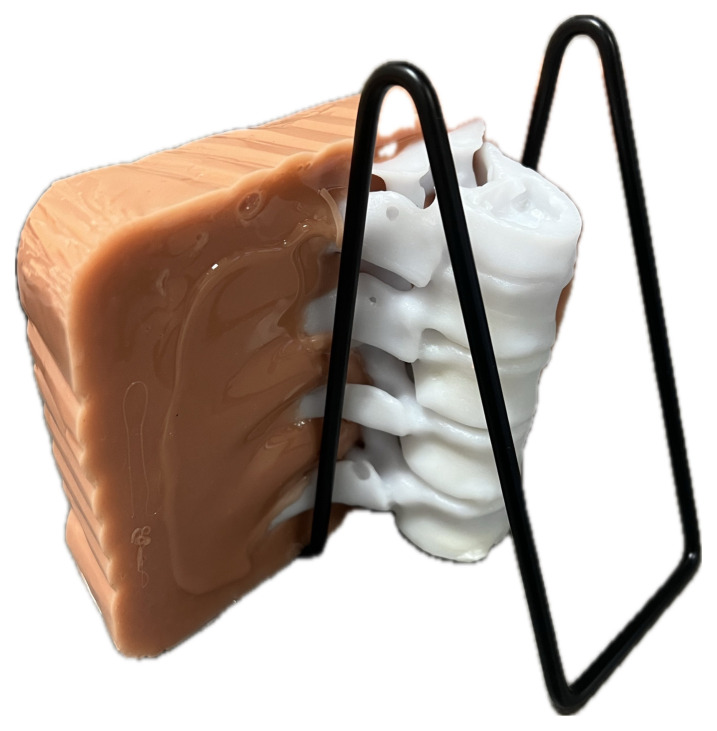
Author’s own image. Final ESP model product.
7. The model was then ready for use. The procedure on the model was conducted on the side covered completely with ballistics gel.[Fig f6-jetem-10-2-i1]
  **Figure 6 f6-jetem-10-2-i1:**
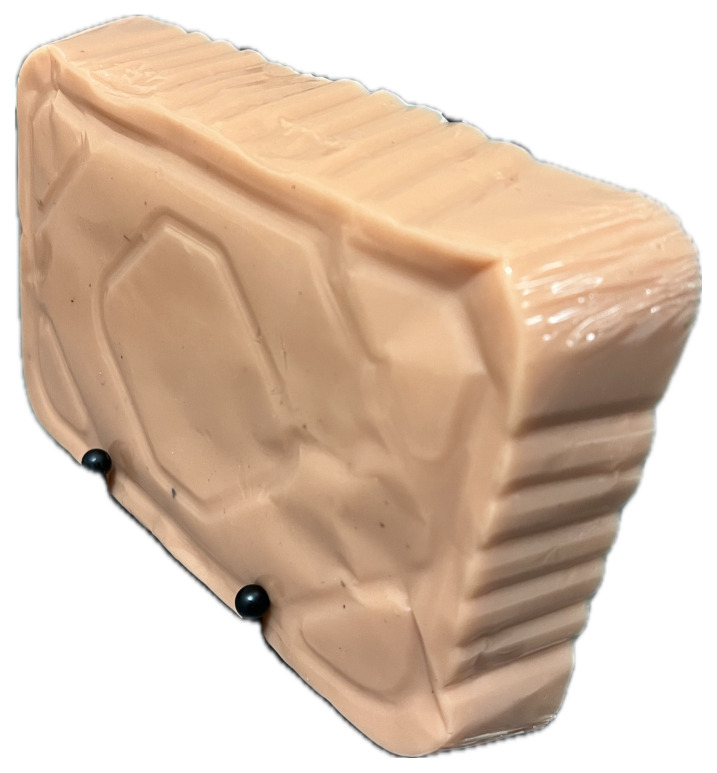
Author’s own image. Proper orientation of the ESP model for usage.
8. During the hands-on training session, the typical set up consisted of the model being placed on a table at approximately the height of how most patients would sit. An ultrasound machine with a linear probe, ultrasound gel, and an echogenic needle were required for the demonstration and practicing of the ESP nerve block.[Fig f7-jetem-10-2-i1]
  **Figure 7 f7-jetem-10-2-i1:**
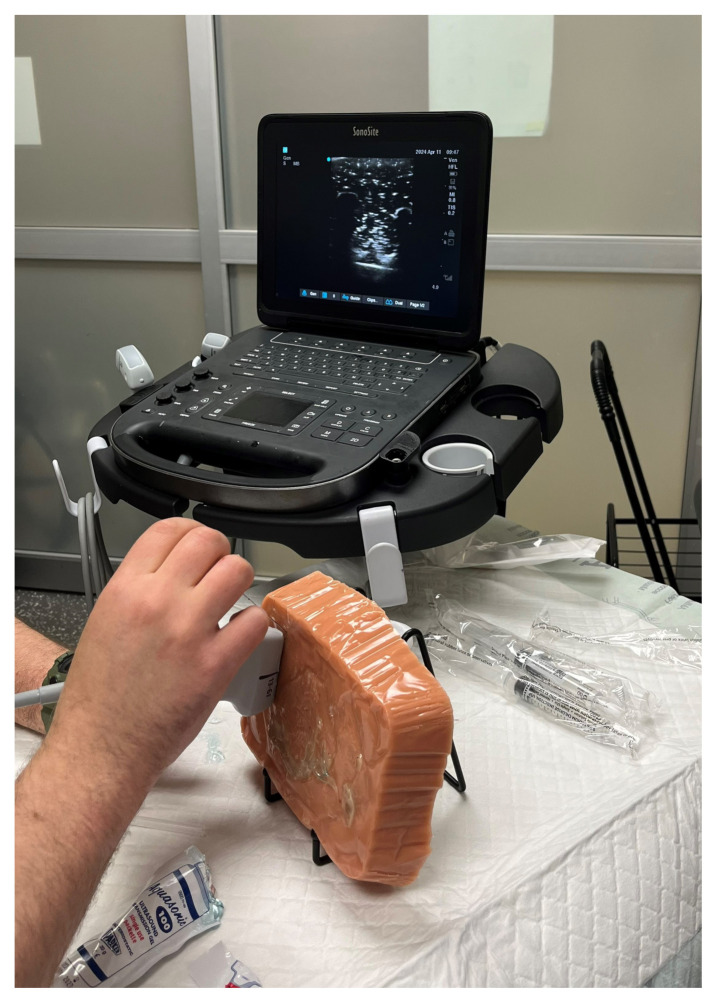
Author’s own image. Demonstration of the ESP model usage.
9. The first step to performing the ESP block on the model involved identifying relevant sonoanatomy and determining the targeted transverse process or rib to administer the medication.[Fig f8-jetem-10-2-i1]
  **Figure 8 f8-jetem-10-2-i1:**
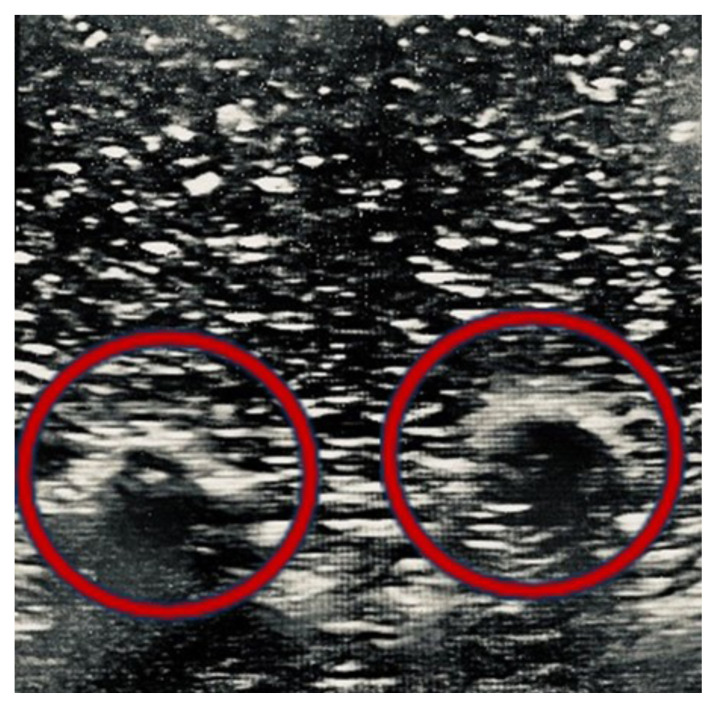
Author’s own image. Ultrasound image of ribs on the created model (red circles).
10. Next, the needle was advanced in-plane until successfully arriving at the targeted rib.[Fig f9-jetem-10-2-i1]
  **Figure 9 f9-jetem-10-2-i1:**
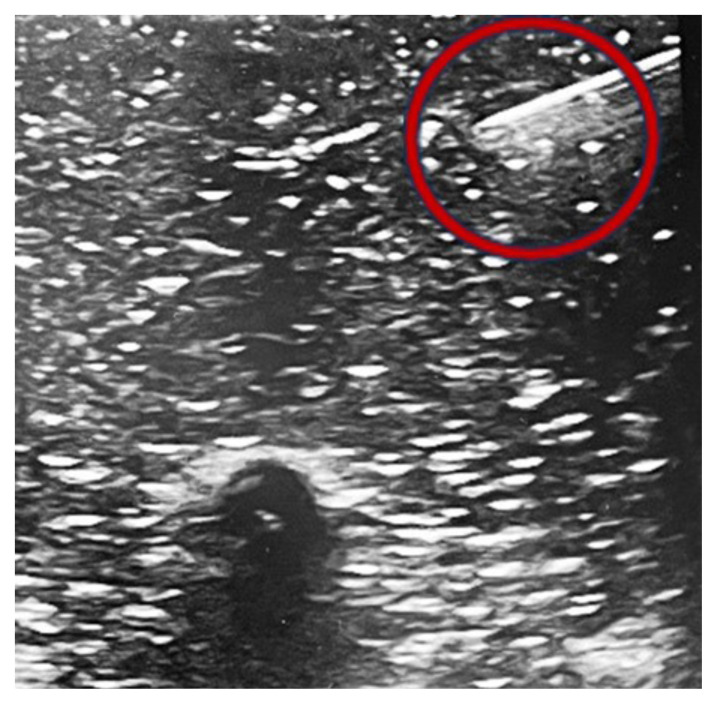
Author’s own image. Reflection of echogenic needle on ultrasound using in-plane technique (red circle).
11. Finally, participants were asked to replicate the procedure again on different TP or rib targets on the model.

### Results and tips for successful implementation

A total of 34 participants attended the training sessions, the majority being ED attending physicians (16/34, 47%) and residents (13/34, 38%), with a small number of medical students, physician assistants, and nurse practitioners (5/34, 15%). Fourteen (14/34, 41%) participants returned completed surveys, of which 50% were residents (7/14, 50%) and 50% were attending physicians (7/14, 50%). No returned surveys were completed by medical students or advanced practice practitioners. The majority (12/14, 86%) of respondents reported no prior experience in performing an ESP block with only 14% (2/14, 14%) reporting performing less than two ESP nerve blocks per year (Table 1).

All respondents (14/14, 100%) agreed or strongly agreed that the training session with the ESP model helped improve their knowledge, confidence, and skills in performing the nerve block. Most respondents (13/14, 93%) agreed or strongly agreed that the created ESP model was similar to a human for performing the ultrasound guided ESP block. All (14/14, 100%) reported feeling confident in their ability to use ultrasound to identify landmarks pertinent to performing the ESP block and most (13/14, 93%) reported confidence in their ability to perform in-plane ultrasound technique (Table 2).

Regarding the relevance of the ESP block, all respondents (14/14, 100%) agreed or strongly agreed that the training material was relevant to their practice in the ED, within their scope of practice, and part of their job as an ED physician. Additionally, all (14/14, 100%) reported that they felt that performing the regional nerve block in the ED will improve patient outcomes and reported an increased likelihood of performing the ESP block on a patient following this training (Table 2).

Lastly, respondents were asked to list any barriers that might inhibit them from performing the ESP block on shift, in addition to any strategies to facilitate ESP block use. Four participants (4/14, 29%) reported barriers to performing an ESP block including time constraints (50%) and patient mobility limitations (50%). Twelve participants (12/14, 86%) reported facilitators to performing ESP blocks, the most common of which was easier access to supplies and assistance with procedure set up (43%), followed by increased education sessions (21%).

After several educational sessions, a limitation was identified with the model. We observed that after frequent punctures of the ballistics gelatin with the echogenic needle, track marks from the needle remained visible in the ultrasound image. To rectify this issue, the model can be disassembled, the ballistics gel re-melted and molded again.

### Associated content

- Crowley, Amelia. PowerPoint: Erector Spinae Plane Block in the ED. 2024.
- Erector Spinae Plane Nerve Block. NYSORA – https://www.nysora.com/erector-spinae-plane-block/

## Supplementary Information



## Figures and Tables

**Figure:**
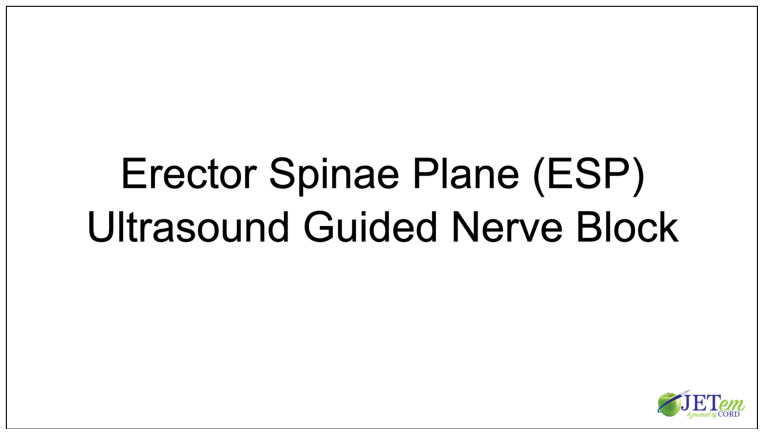
Please see associated PowerPoint file

**Table 1:** Participant Characteristics

Characteristic	Survey Respondents n = 14
Title	
PGY1	3 (21%)
PGY2	2 (14%)
PGY3	2 (14%)
Attending Physician	7 (50%)
Experience with Performing ESP Blocks	
Never Performed an ESP Block	12 (86%)
Perform less than two ESP Blocks per Year	2 (14%)

**Table 2:** Post-Training Survey Responses

Question	Response				
	*Strongly Disagree*	*Disagree*	*Neither Agree nor Disagree*	*Agree*	*Strongly Agree*
Overall, I found this training session increased my confidence in performing an ultrasound-guided ESP block in the ED.				6 (43%)	8 (57%)

Overall, I found this training session improved my knowledge in performing ultrasound-guided ESP blocks in the ED.				5 (36%)	9 (64%)

I feel I have gained adequate skills to perform an ESP block in the ED.				7 (50%)	7 (50%)

Thinking about the created spine model, I found it was similar to a human for performing ultrasound guided ESP blocks in the ED.			1 (7%)	11 (79%)	2 (14%)

I am confident in my ability to use ultrasound to identify landmarks pertinent to performing an ESP block.				9 (64%)	5 (36%)

I am confident in my ability to perform in-plane techniques.			1 (7%)	8 (57%)	5 (36%)

This material was relevant to my practice in the ED, is within my scope of practice, and part of my job as an ED physician.				5 (36%)	9 (64%)

I am confident in my ability to identify and manage potential complications that could arise as a result of the ESP block.			1 (7%)	9 (64%)	4 (29%)

I feel that performing regional nerve blocks in the ED will improve patient outcomes.				8 (57%)	6 (43%)

After this training session, I am now likely to perform an ESP block on a patient in the ED.				10 (71%)	4 (29%)
