# Supplementary material for: Innovative Ultrasound-Guided Erector Spinae Plane Nerve Block Model for Training Emergency Medicine Physicians
Source: J Educ Teach Emerg Med. 2025 Apr 30;10(2):I1–I10. doi: 10.21980/J8PW7D (PMC12054114; doi:10.21980/J8PW7D)
Supplement: Supplementary file 1 [file 10-2-I1-supp1.pptx]

## Slide 1
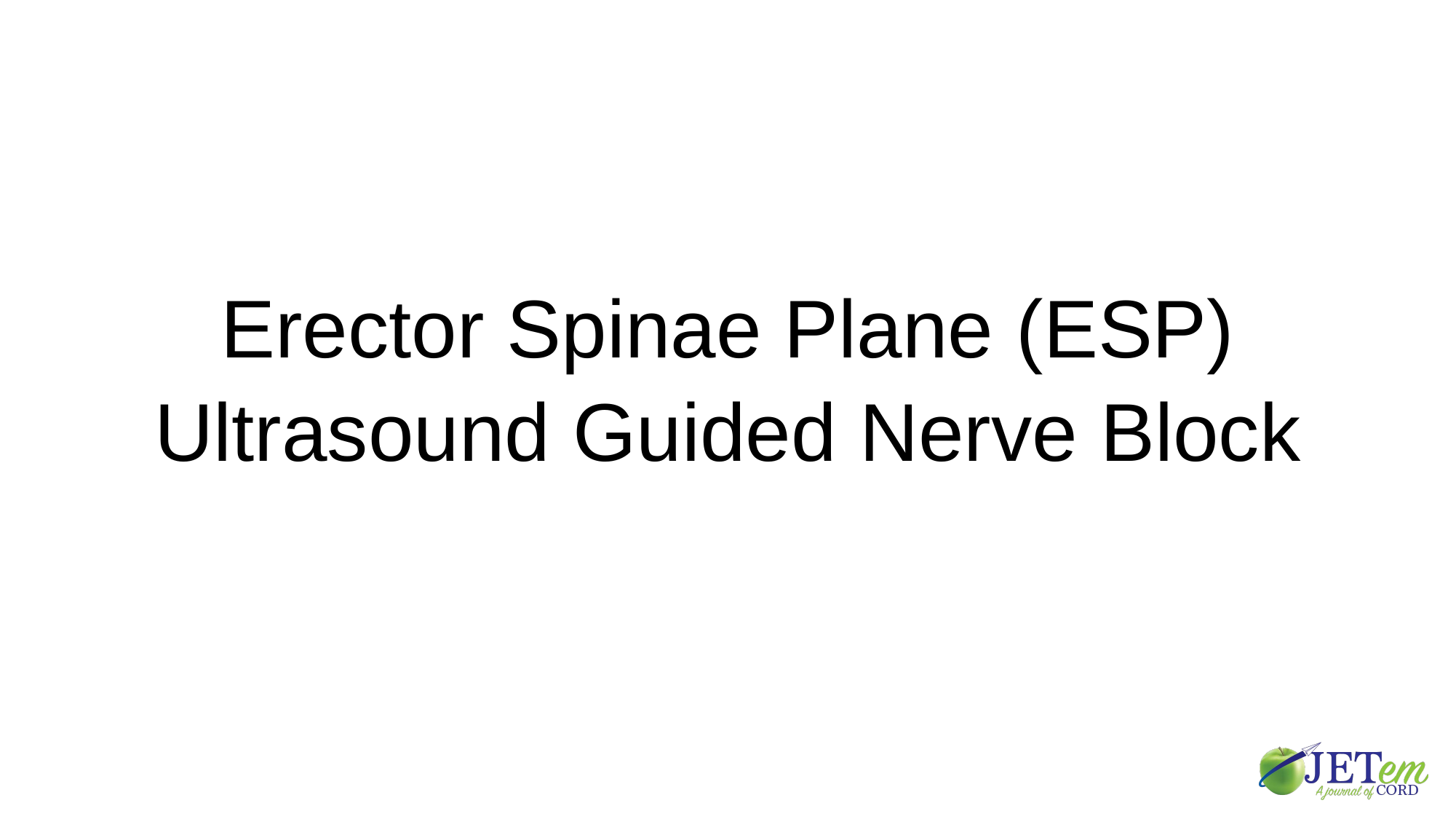

Erector Spinae Plane (ESP)
Ultrasound Guided Nerve Block

## Slide 2
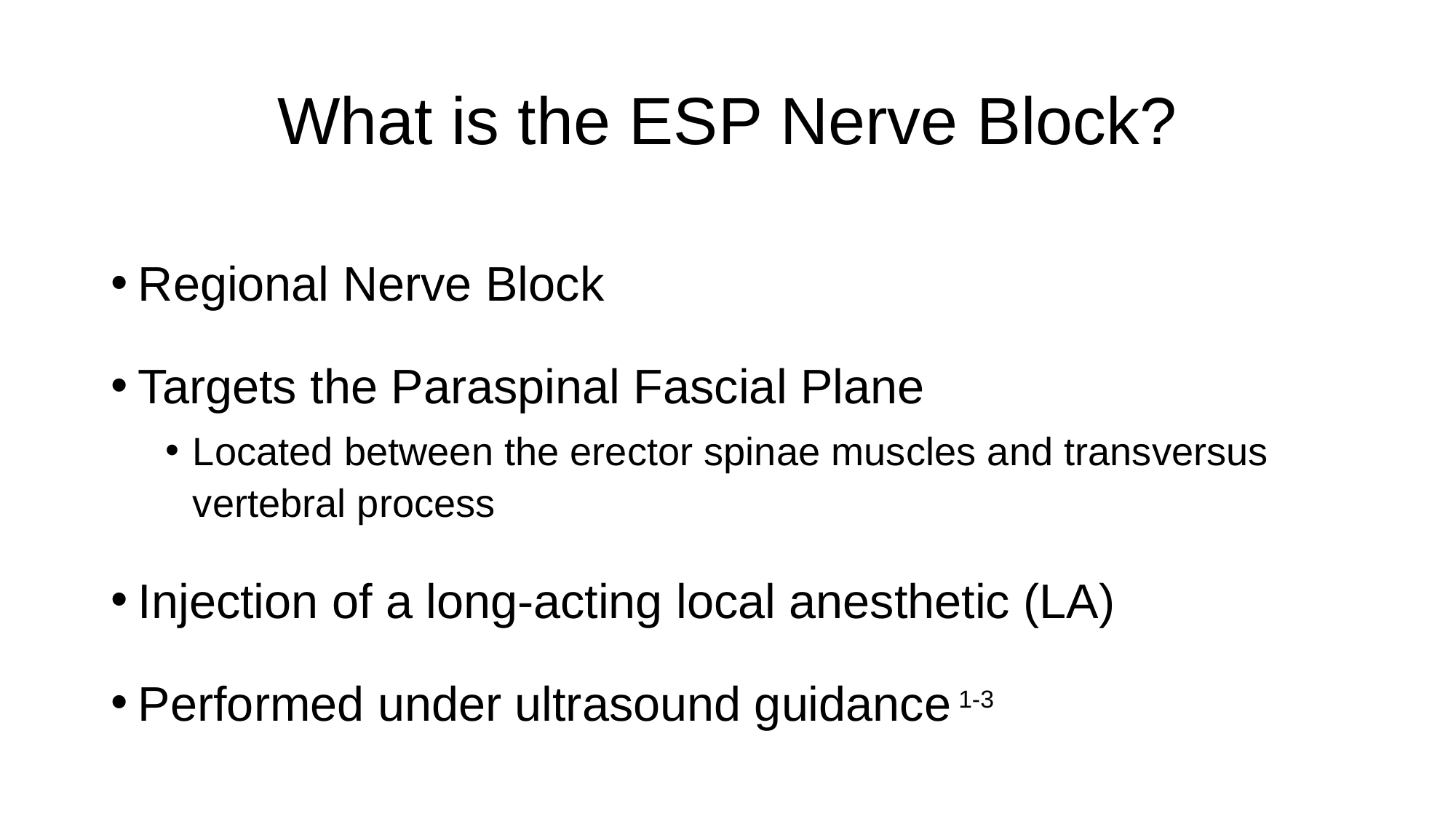

# What is the ESP Nerve Block?
Regional Nerve Block
Targets the Paraspinal Fascial Plane
Located between the erector spinae muscles and transversus vertebral process
Injection of a long-acting local anesthetic (LA)
Performed under ultrasound guidance 1-3

## Slide 3
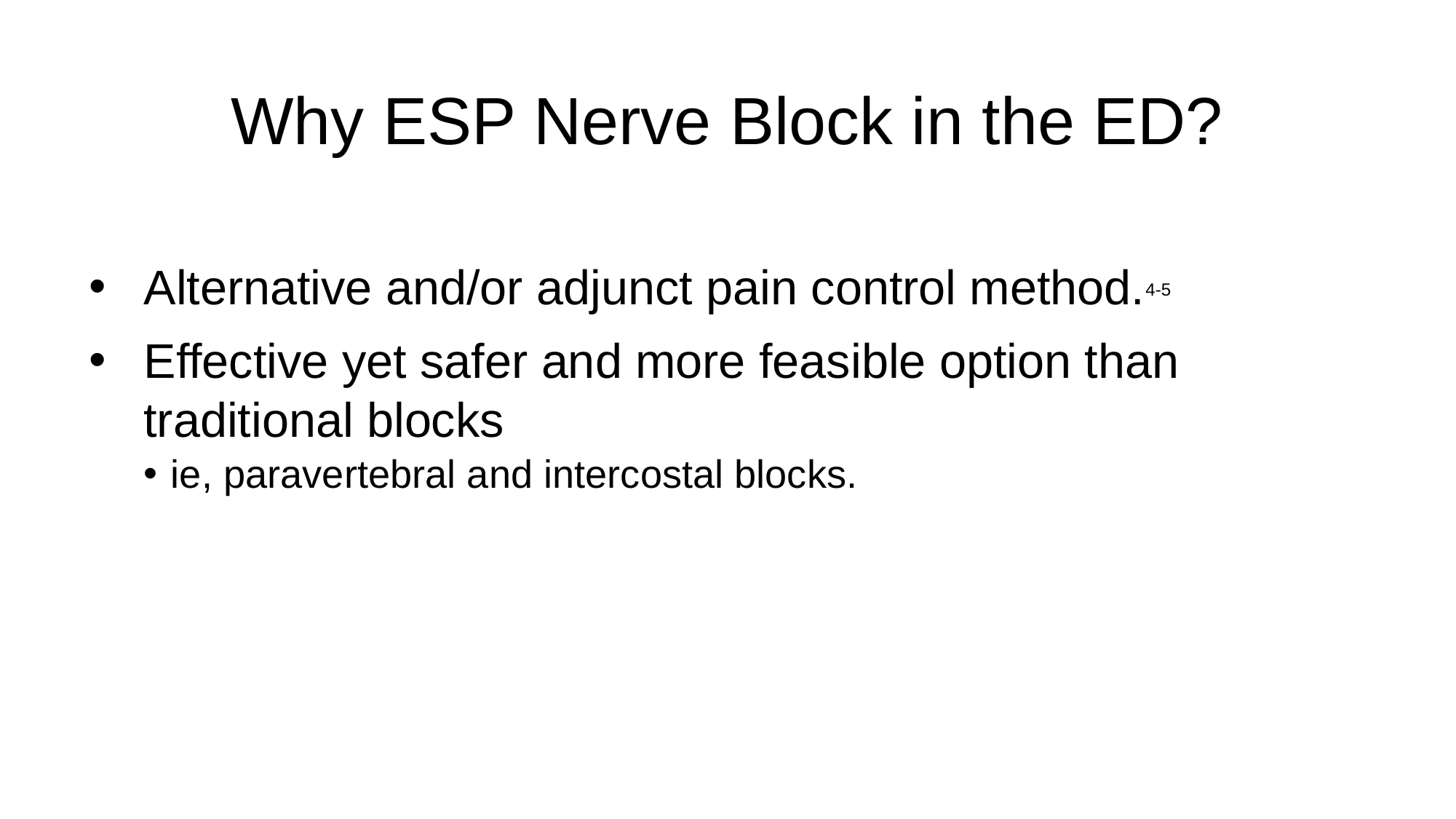

# Why ESP Nerve Block in the ED?
Alternative and/or adjunct pain control method.4-5
Effective yet safer and more feasible option than traditional blocks
ie, paravertebral and intercostal blocks.

## Slide 4
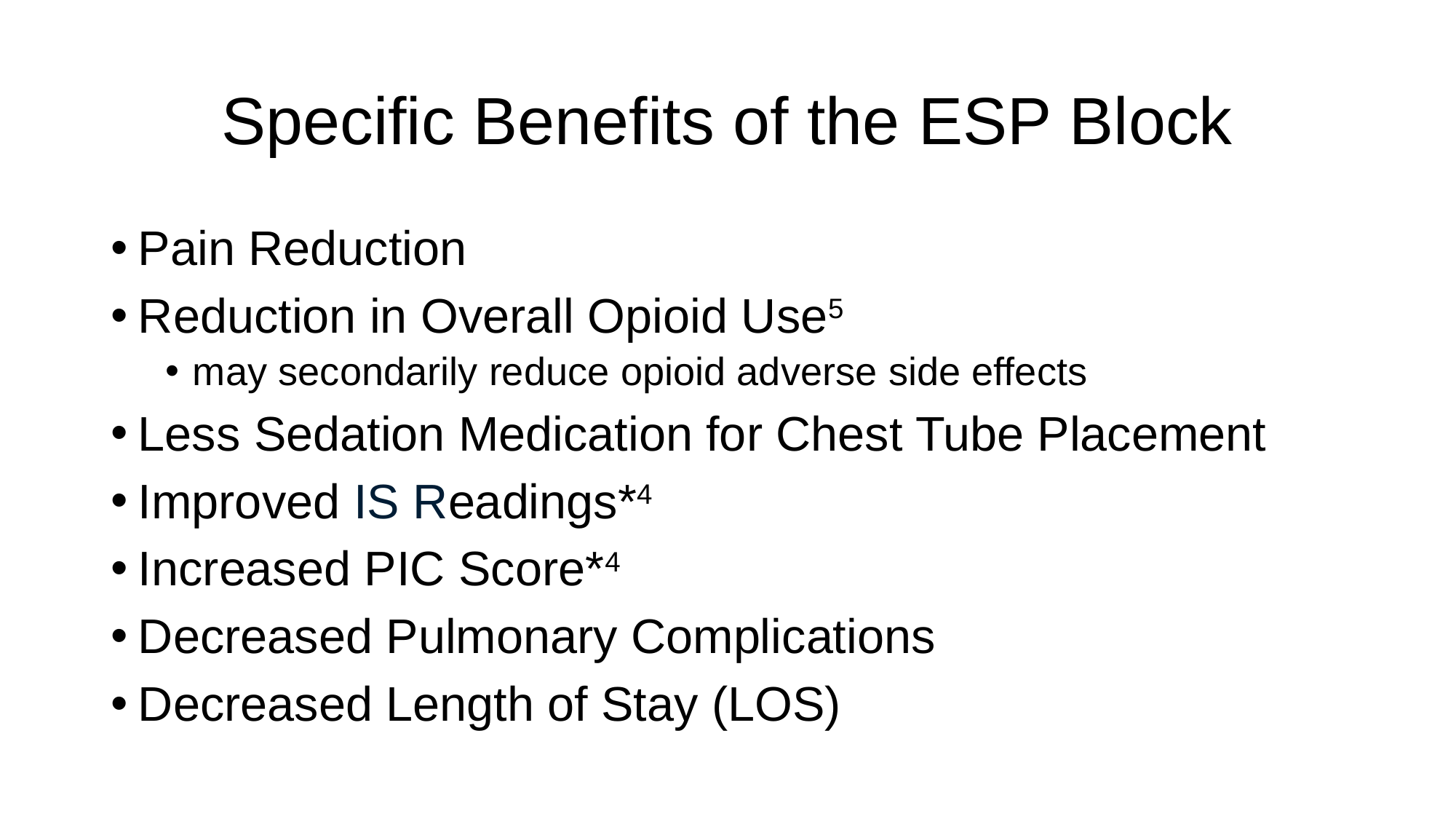

# Specific Benefits of the ESP Block
Pain Reduction
Reduction in Overall Opioid Use5
may secondarily reduce opioid adverse side effects
Less Sedation Medication for Chest Tube Placement
Improved IS Readings*4
Increased PIC Score*4
Decreased Pulmonary Complications
Decreased Length of Stay (LOS)

## Slide 5
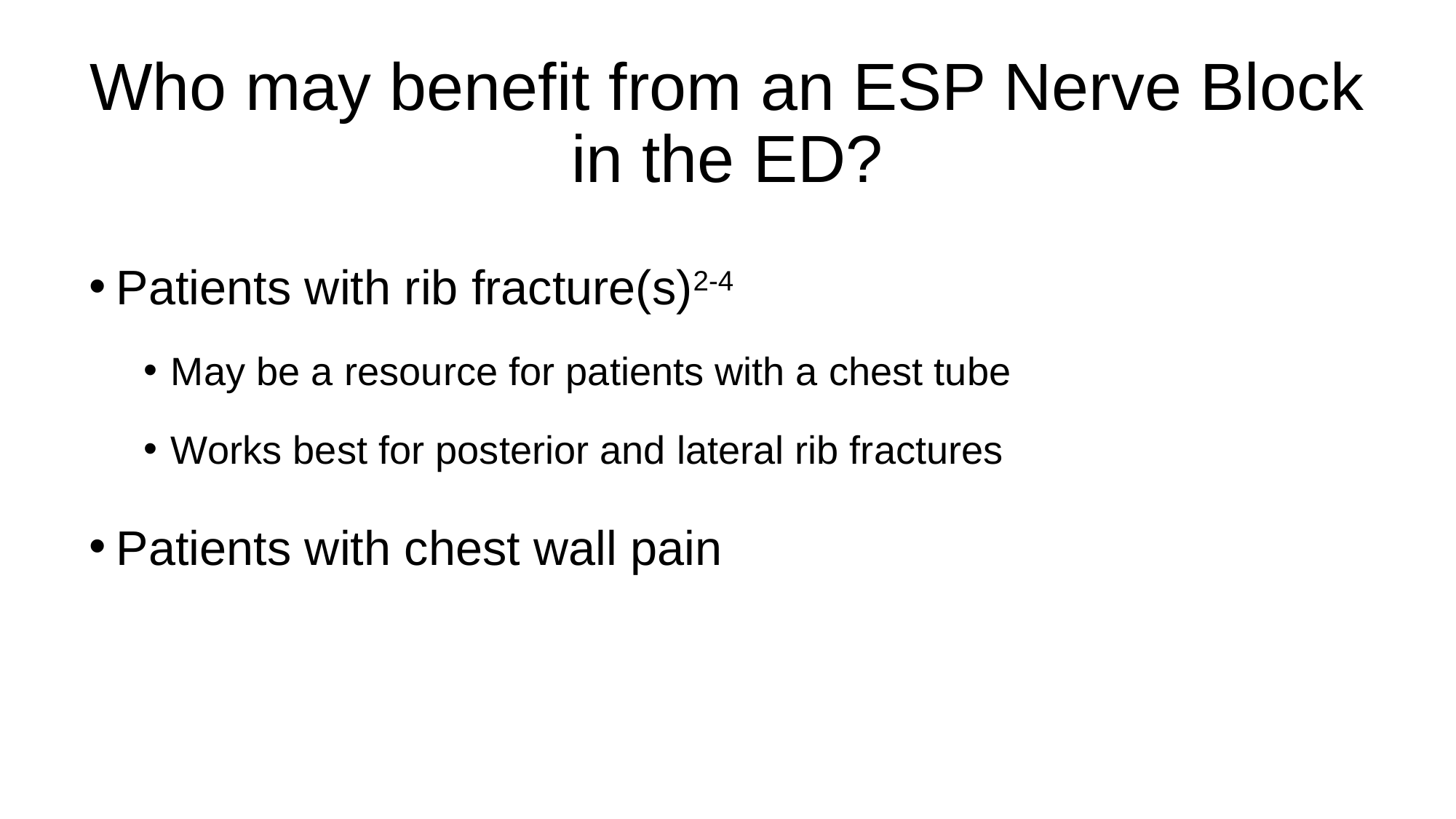

# Who may benefit from an ESP Nerve Block in the ED?
Patients with rib fracture(s)2-4
May be a resource for patients with a chest tube
Works best for posterior and lateral rib fractures
Patients with chest wall pain

## Slide 6
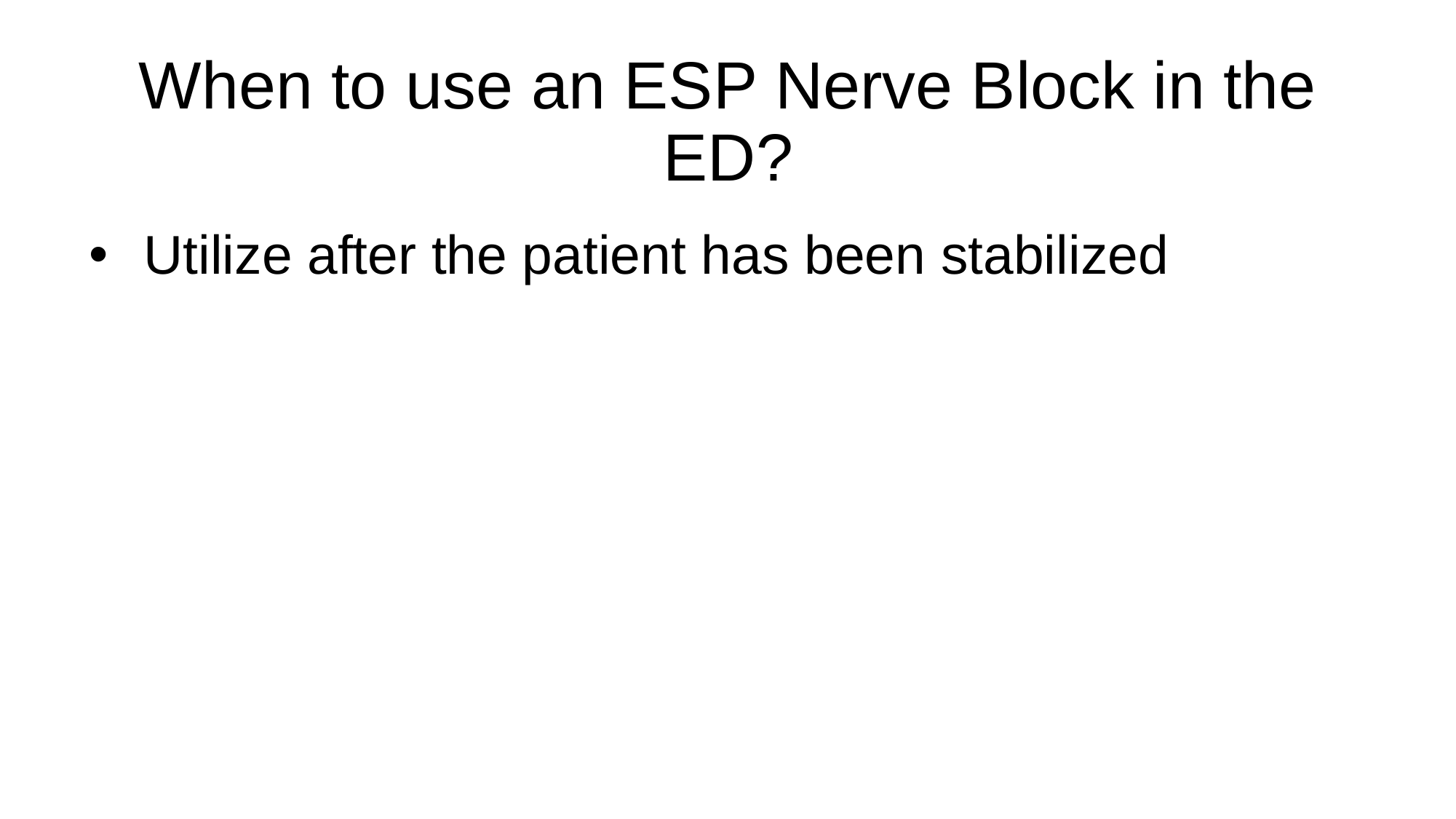

# When to use an ESP Nerve Block in the ED?
Utilize after the patient has been stabilized

## Slide 7
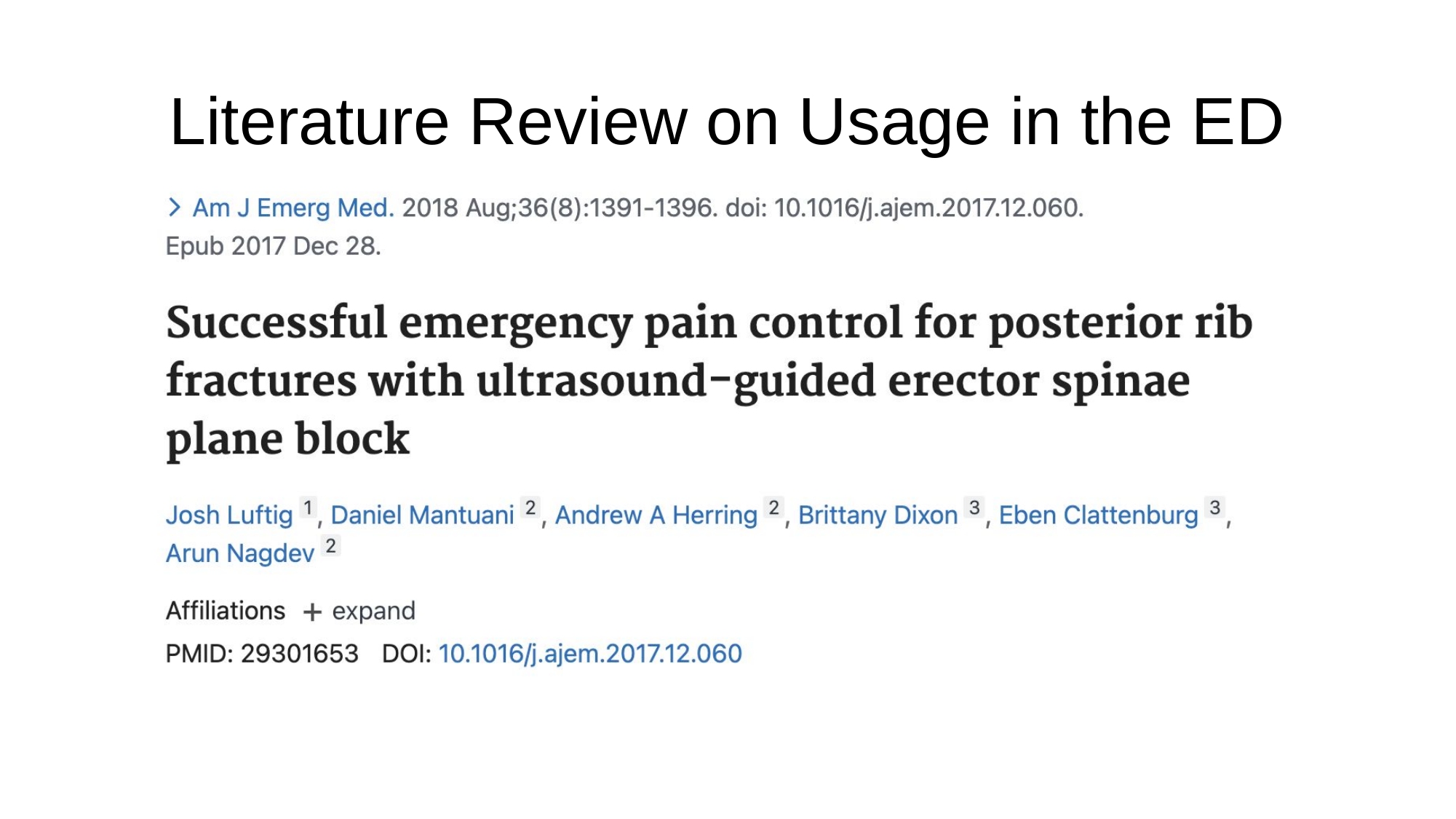

# Literature Review on Usage in the ED

## Slide 8
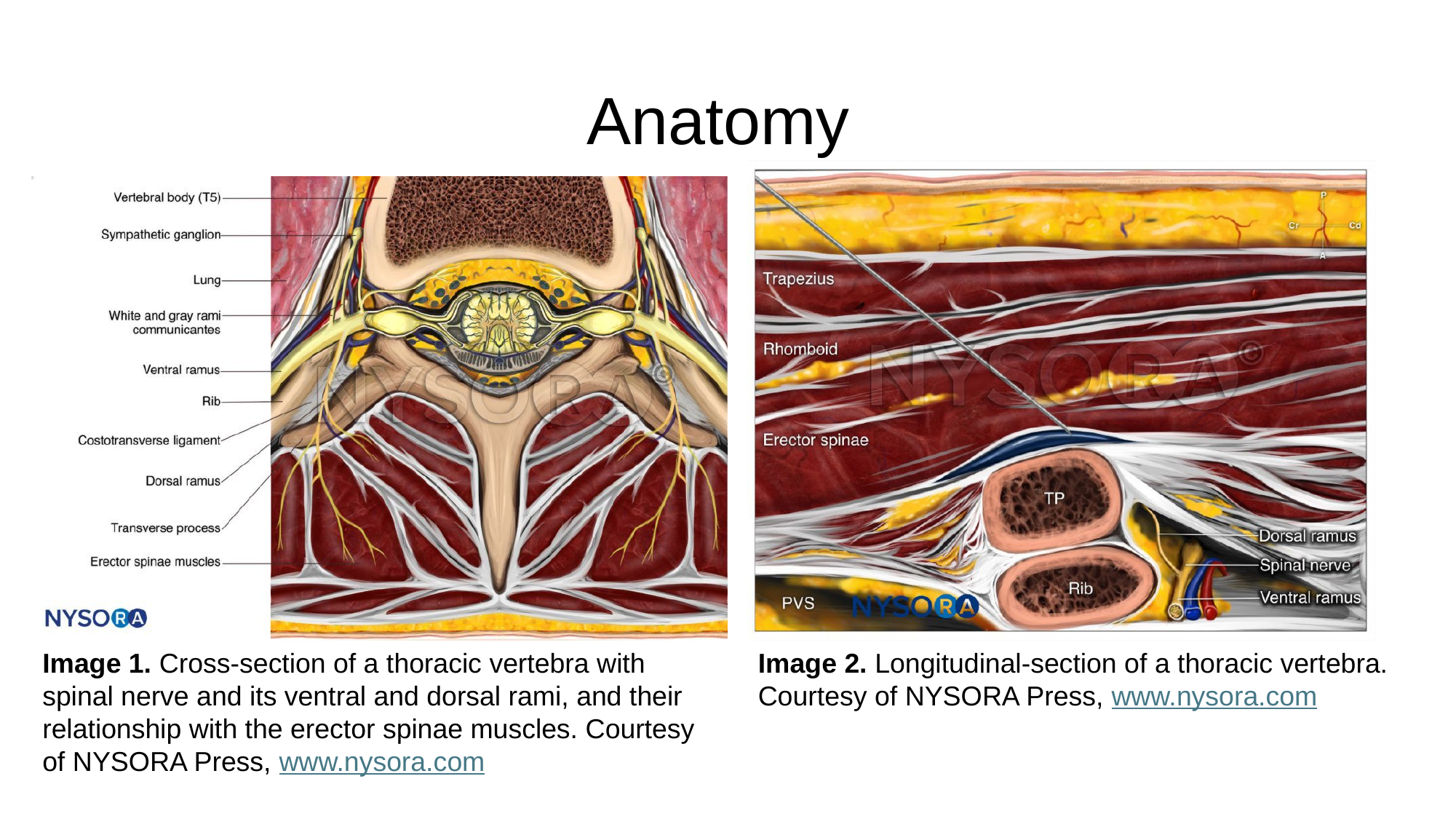

# Anatomy
Image 1. Cross-section of a thoracic vertebra with spinal nerve and its ventral and dorsal rami, and their relationship with the erector spinae muscles. Courtesy of NYSORA Press, www.nysora.com
Image 2. Longitudinal-section of a thoracic vertebra. Courtesy of NYSORA Press, www.nysora.com

## Slide 9
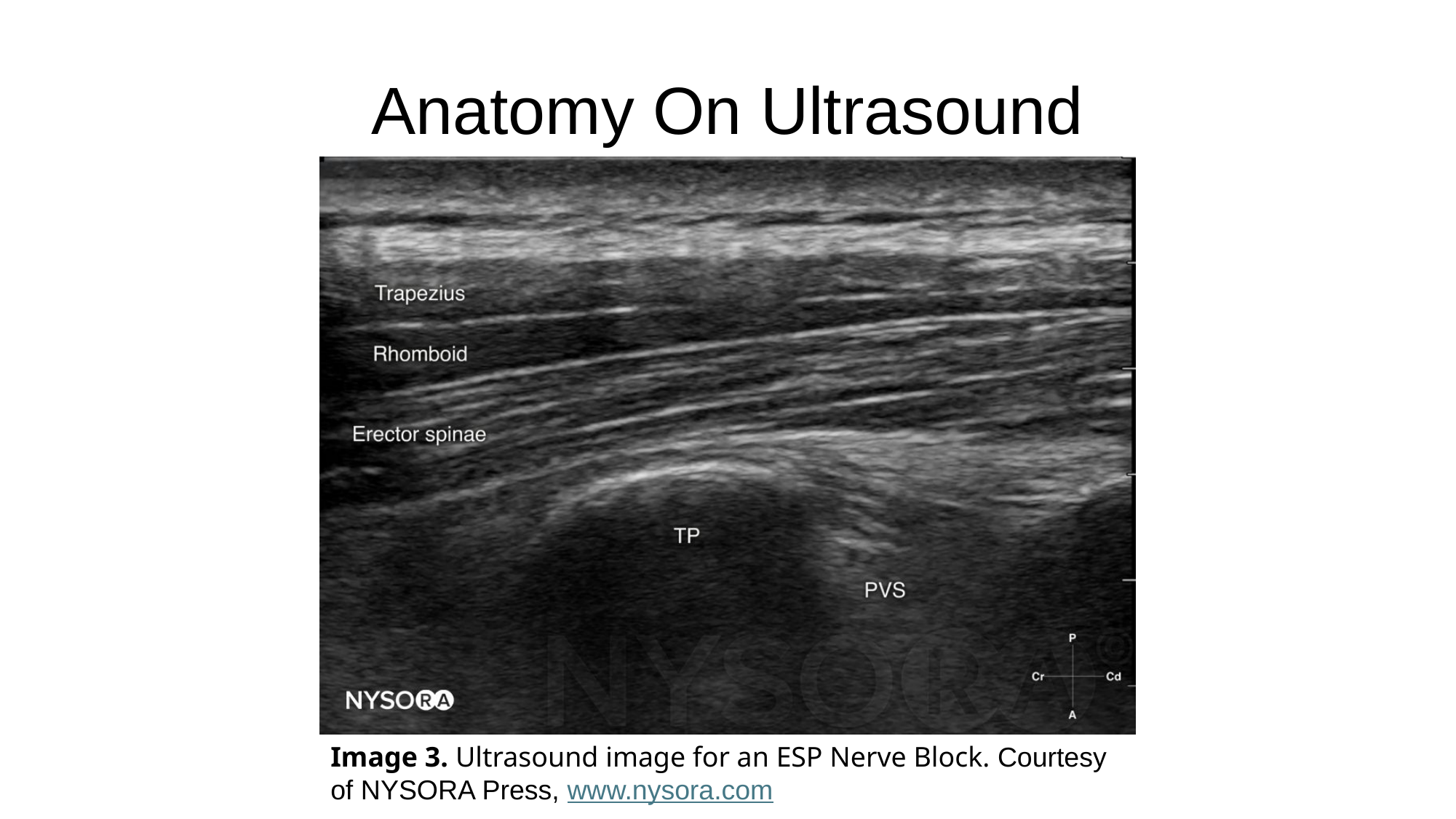

# Anatomy On Ultrasound
Image 3. Ultrasound image for an ESP Nerve Block. Courtesy of NYSORA Press, www.nysora.com

## Slide 10
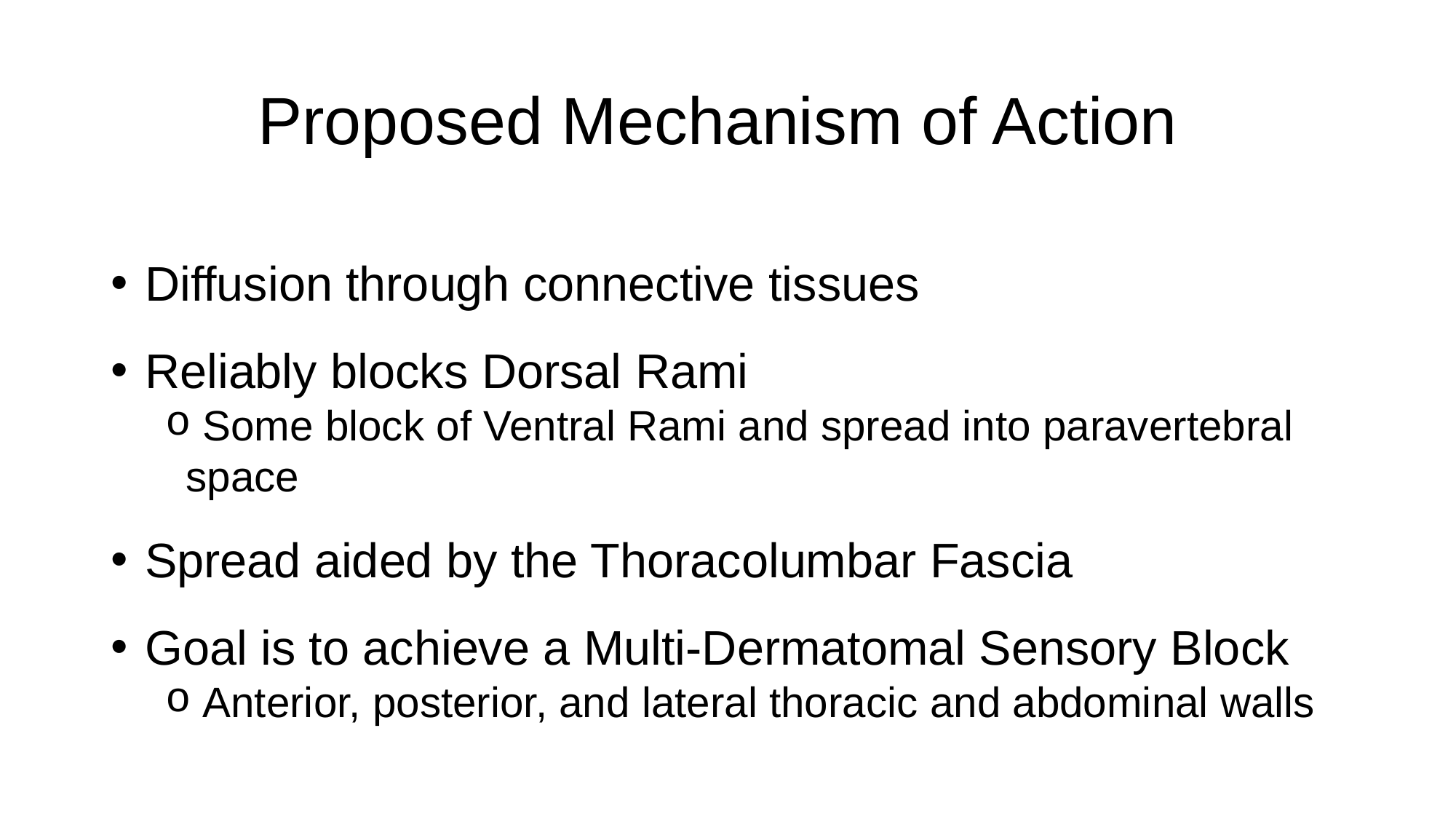

# Proposed Mechanism of Action
 Diffusion through connective tissues
 Reliably blocks Dorsal Rami
 Some block of Ventral Rami and spread into paravertebral space
 Spread aided by the Thoracolumbar Fascia
 Goal is to achieve a Multi-Dermatomal Sensory Block
 Anterior, posterior, and lateral thoracic and abdominal walls

## Slide 11
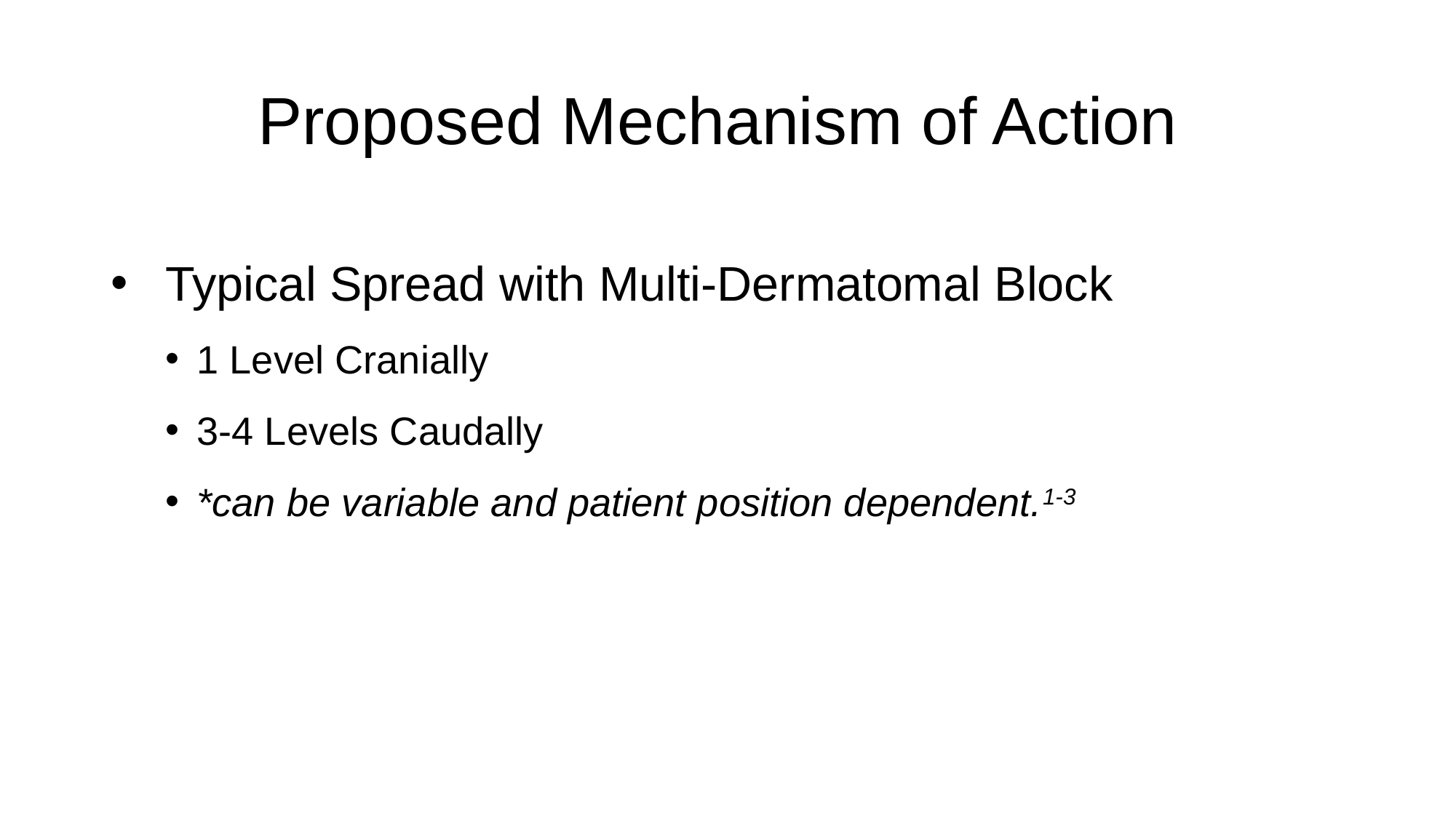

# Proposed Mechanism of Action
Typical Spread with Multi-Dermatomal Block
 1 Level Cranially
 3-4 Levels Caudally
 *can be variable and patient position dependent.1-3

## Slide 12
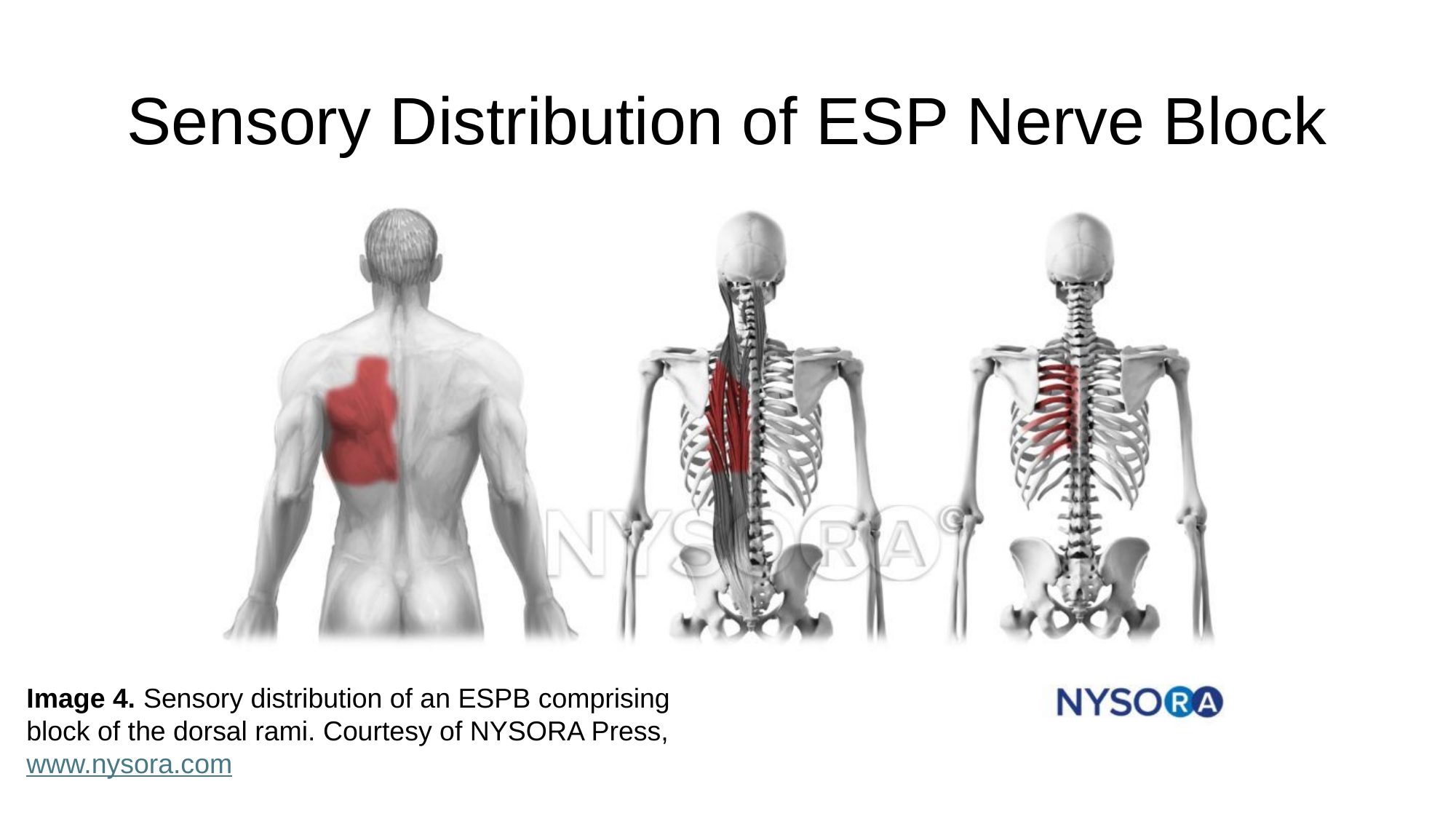

# Sensory Distribution of ESP Nerve Block
Image 4. Sensory distribution of an ESPB comprising block of the dorsal rami. Courtesy of NYSORA Press, www.nysora.com

## Slide 13
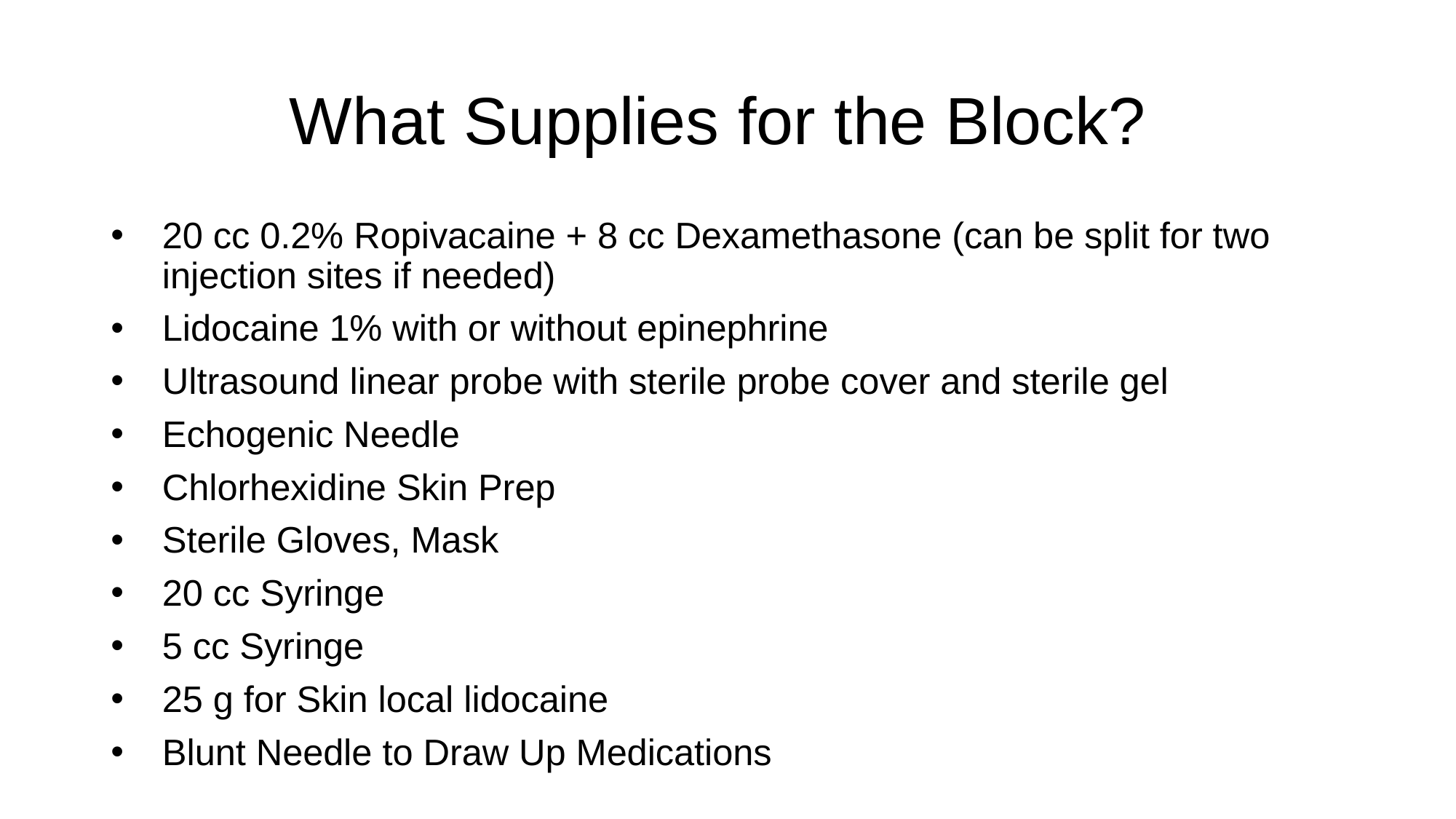

# What Supplies for the Block?
20 cc 0.2% Ropivacaine + 8 cc Dexamethasone (can be split for two injection sites if needed)
Lidocaine 1% with or without epinephrine
Ultrasound linear probe with sterile probe cover and sterile gel
Echogenic Needle
Chlorhexidine Skin Prep
Sterile Gloves, Mask
20 cc Syringe
5 cc Syringe
25 g for Skin local lidocaine
Blunt Needle to Draw Up Medications

## Slide 14
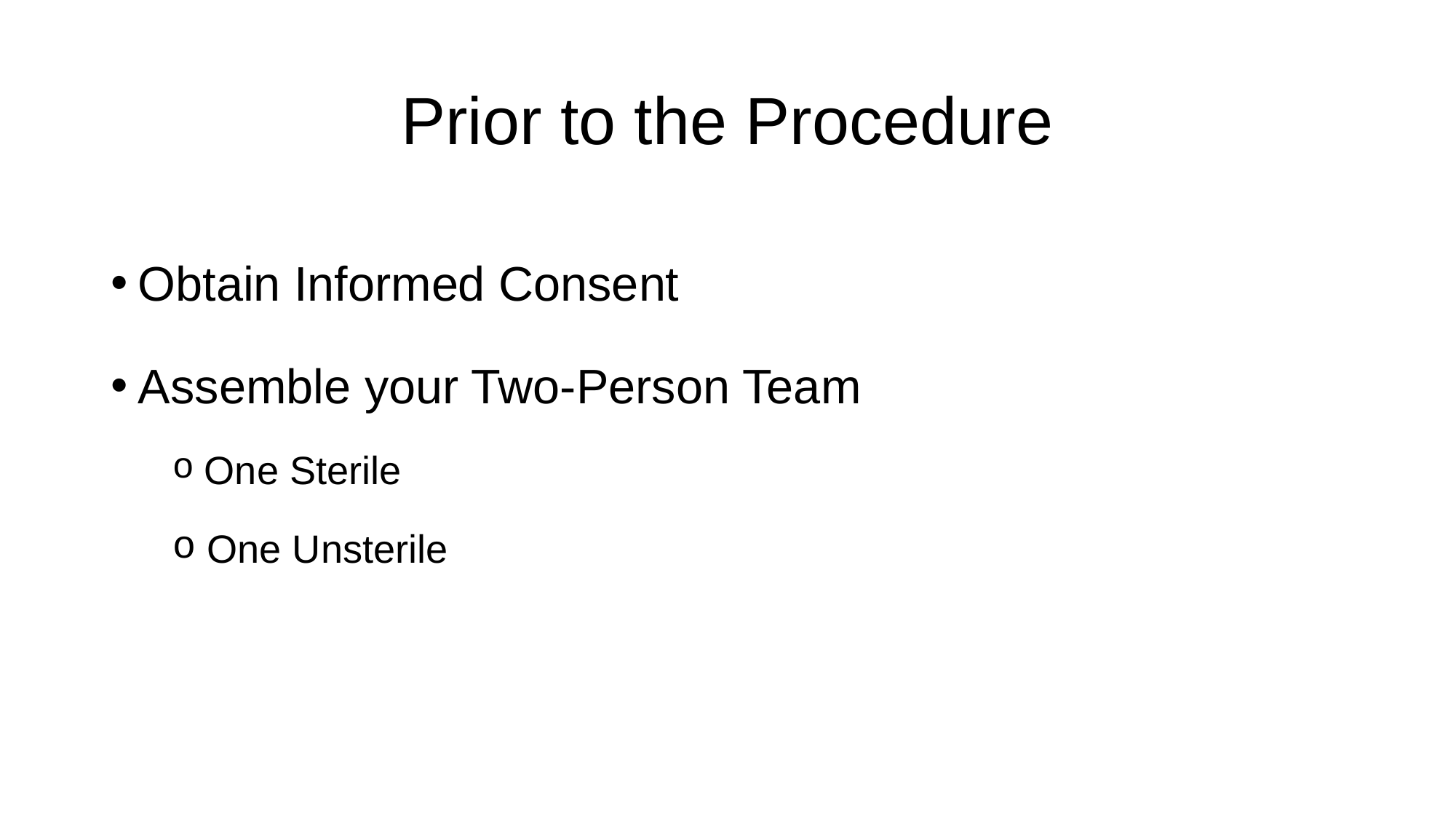

# Prior to the Procedure
Obtain Informed Consent
Assemble your Two-Person Team
 One Sterile
 One Unsterile

## Slide 15
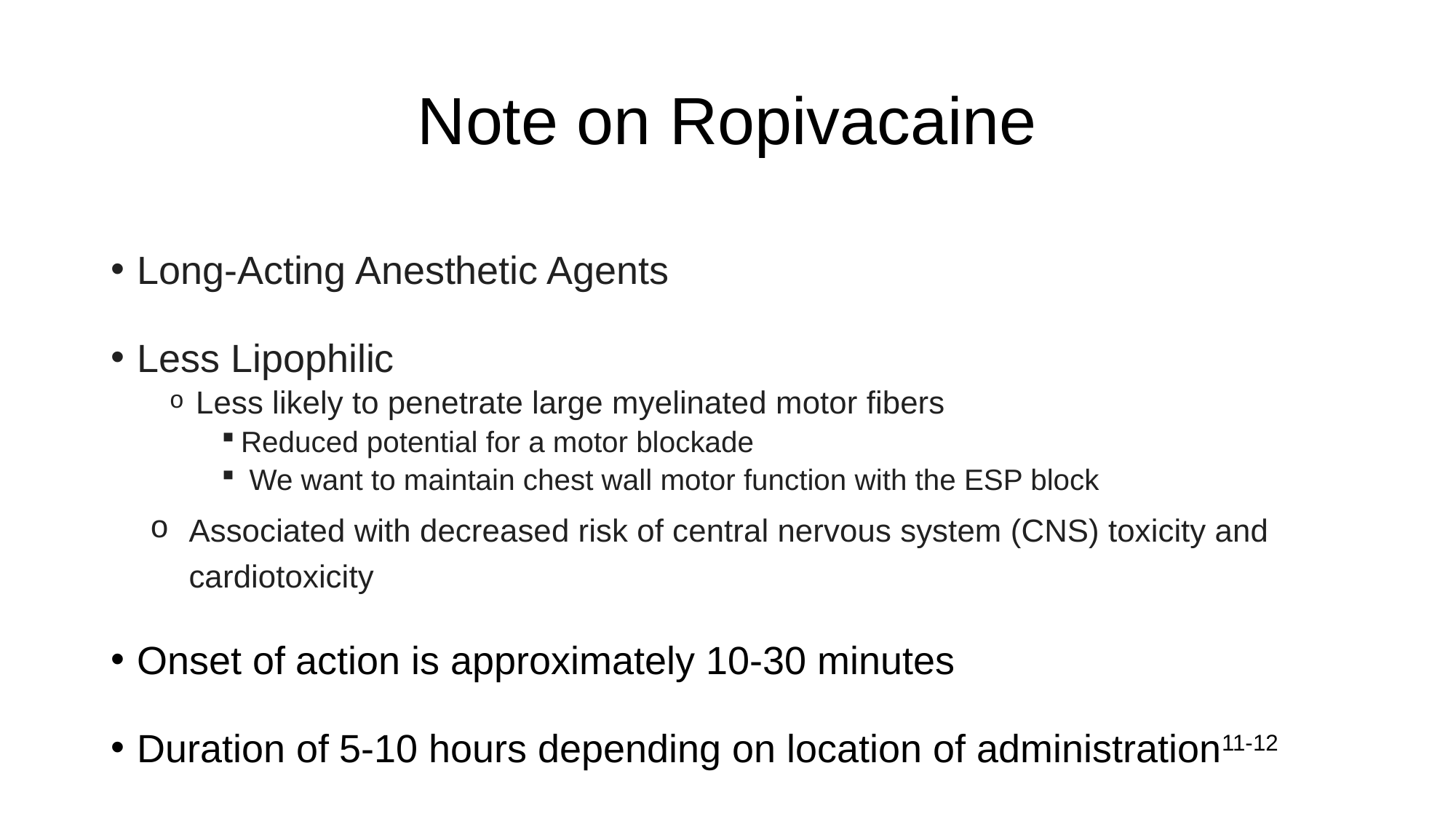

# Note on Ropivacaine
Long-Acting Anesthetic Agents
Less Lipophilic
 Less likely to penetrate large myelinated motor fibers
Reduced potential for a motor blockade
 We want to maintain chest wall motor function with the ESP block
Associated with decreased risk of central nervous system (CNS) toxicity and cardiotoxicity
Onset of action is approximately 10-30 minutes
Duration of 5-10 hours depending on location of administration11-12

## Slide 16
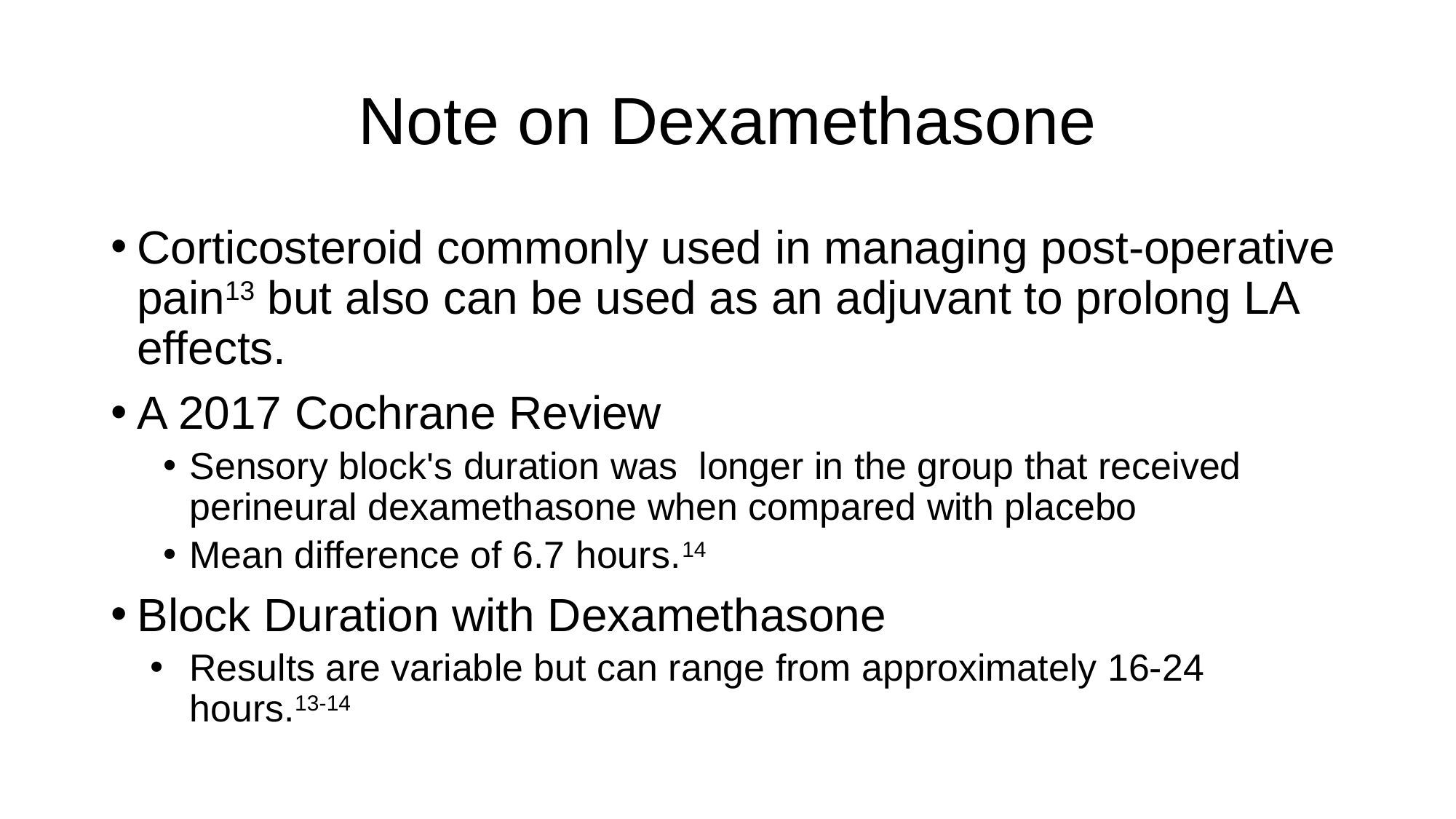

# Note on Dexamethasone
Corticosteroid commonly used in managing post-operative pain13 but also can be used as an adjuvant to prolong LA effects.
A 2017 Cochrane Review
Sensory block's duration was  longer in the group that received perineural dexamethasone when compared with placebo
Mean difference of 6.7 hours.14
Block Duration with Dexamethasone
Results are variable but can range from approximately 16-24 hours.13-14

## Slide 17
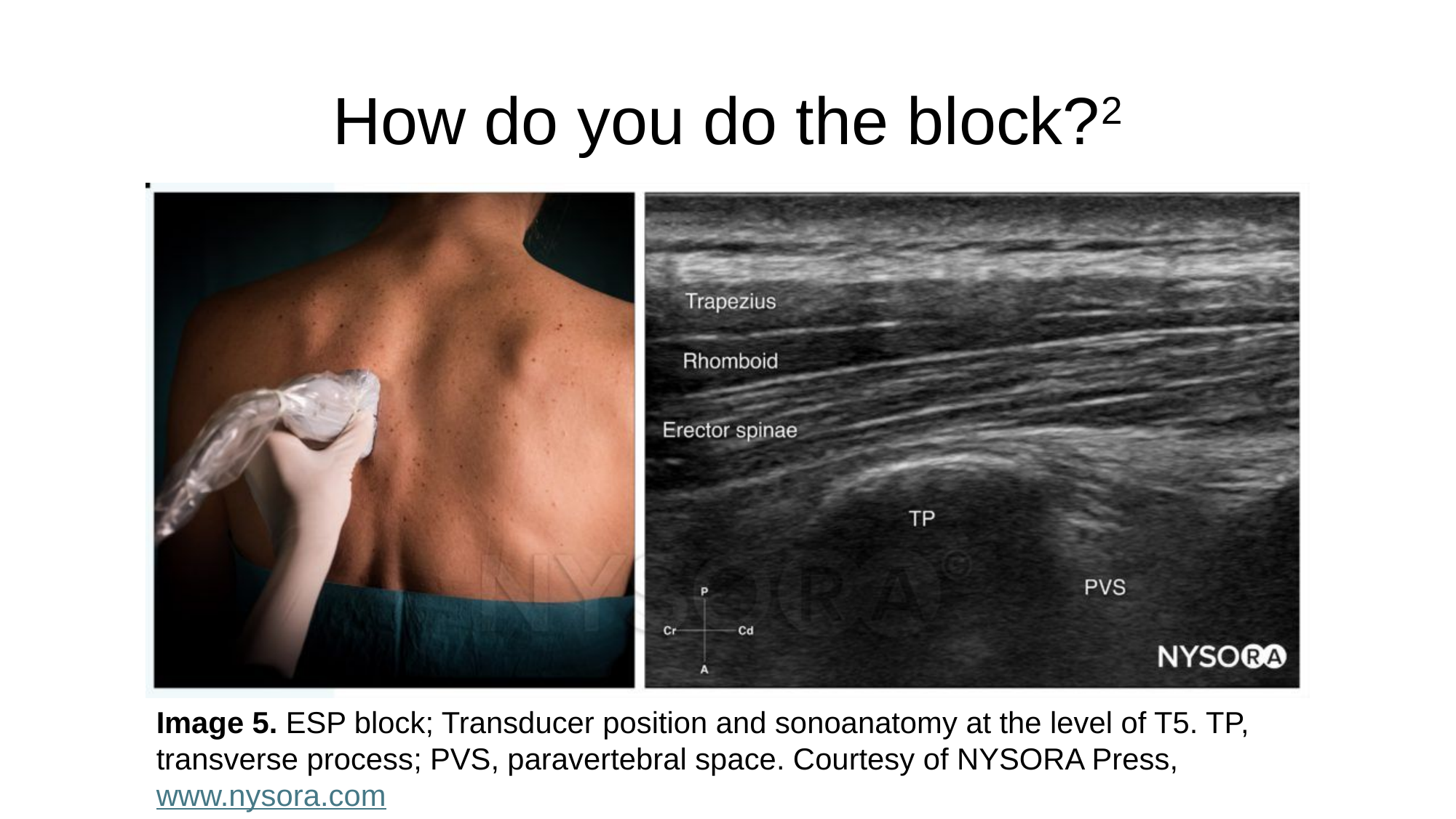

# How do you do the block?2
Image 5. ESP block; Transducer position and sonoanatomy at the level of T5. TP, transverse process; PVS, paravertebral space. Courtesy of NYSORA Press, www.nysora.com

## Slide 18
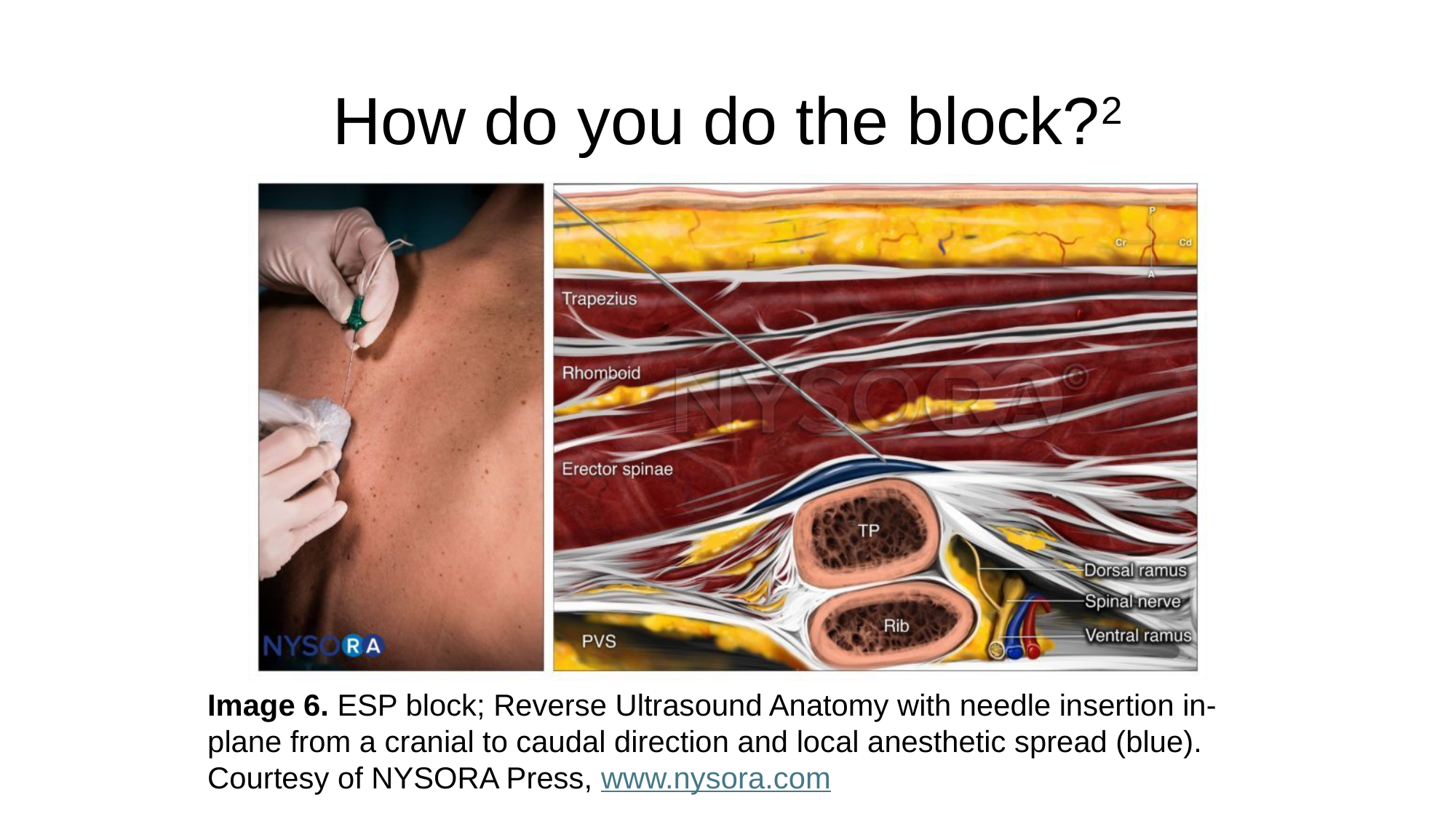

# How do you do the block?2
Image 6. ESP block; Reverse Ultrasound Anatomy with needle insertion in-plane from a cranial to caudal direction and local anesthetic spread (blue). Courtesy of NYSORA Press, www.nysora.com

## Slide 19
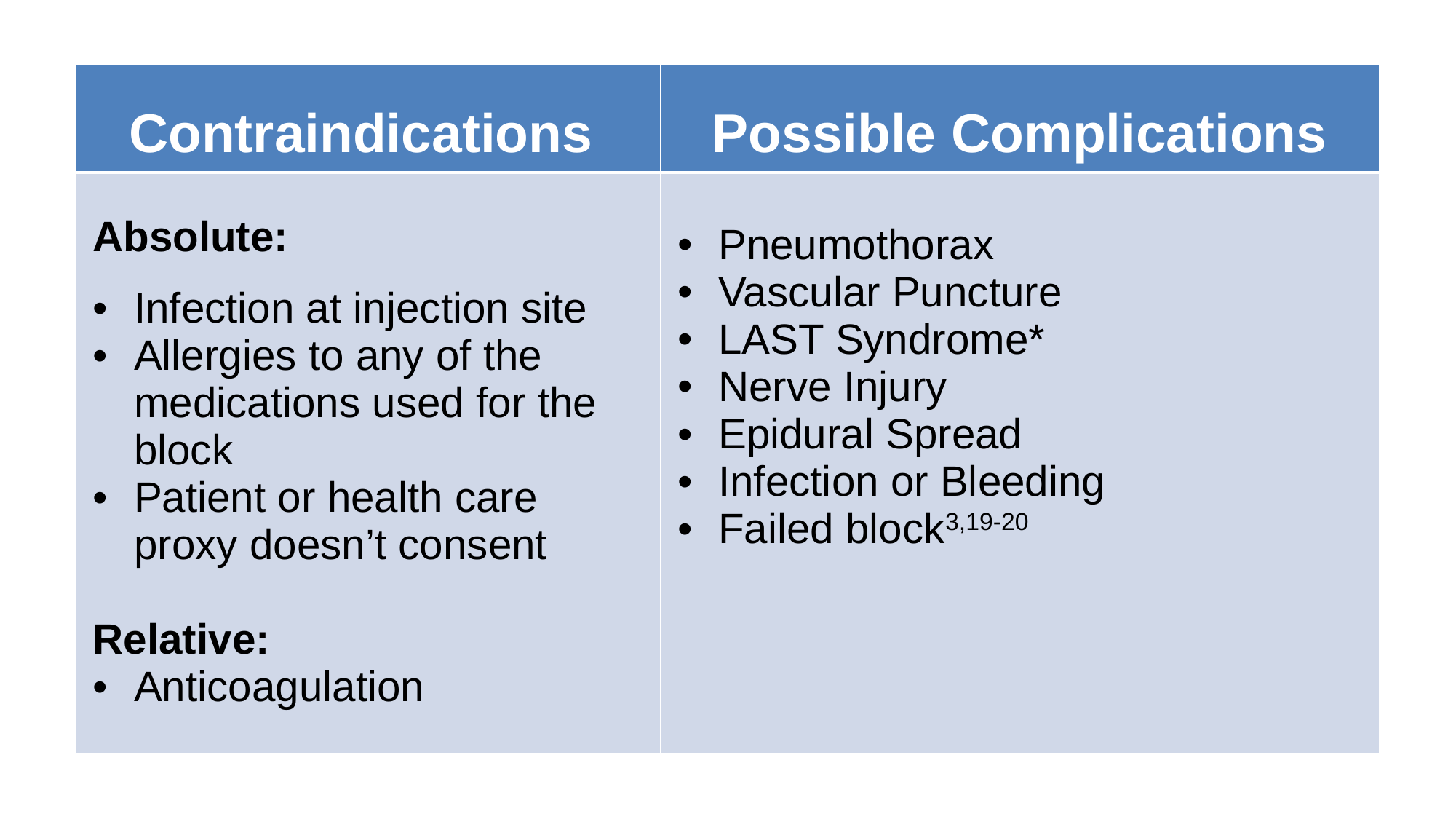

| Contraindications | Possible Complications |
| --- | --- |
| Absolute: Infection at injection site Allergies to any of the medications used for the block Patient or health care proxy doesn’t consent Relative:  Anticoagulation | Pneumothorax Vascular Puncture LAST Syndrome\* Nerve Injury Epidural Spread Infection or Bleeding Failed block3,19-20 |

## Slide 20
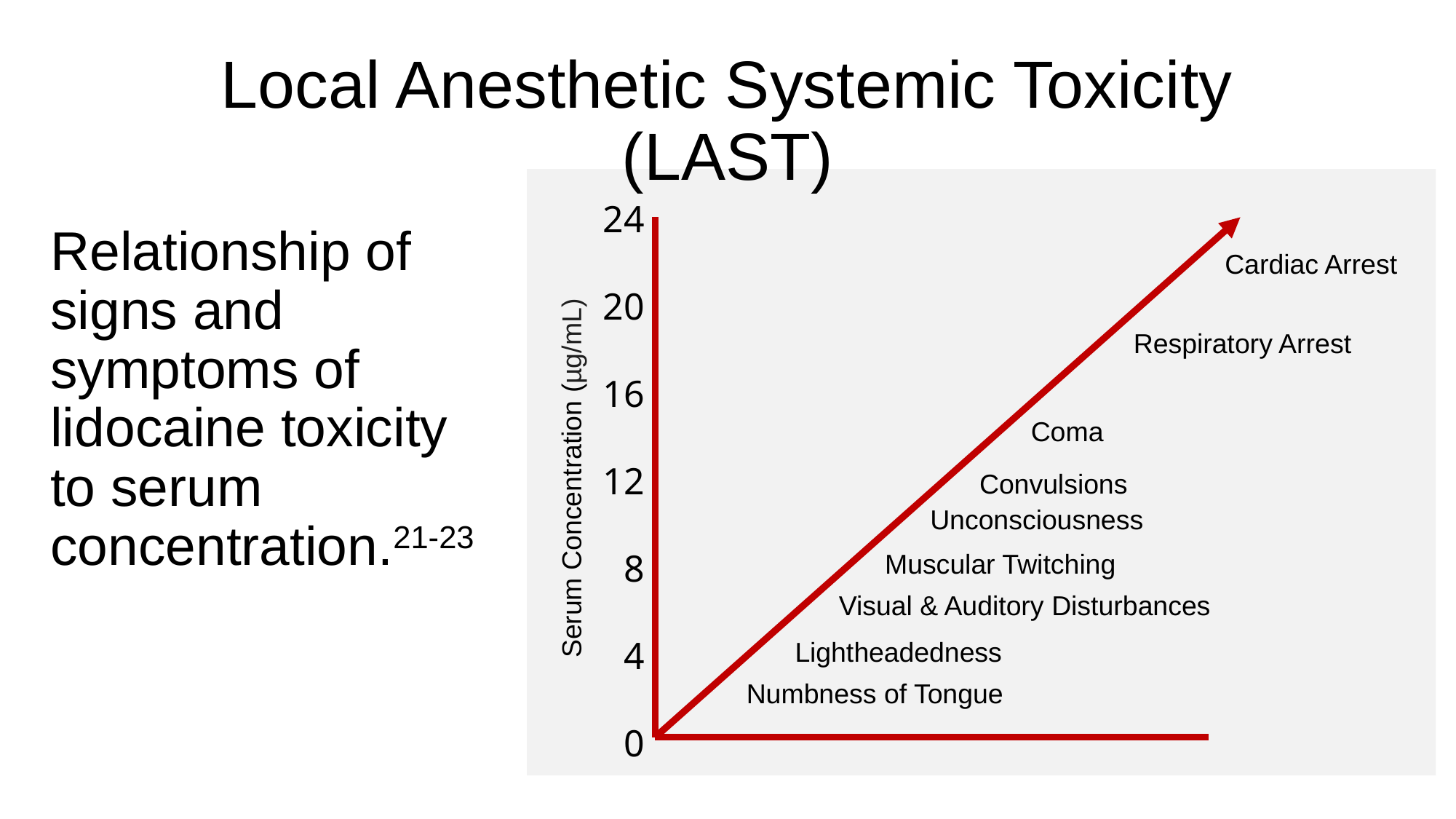

# Local Anesthetic Systemic Toxicity (LAST)
Cardiac Arrest
Respiratory Arrest
Coma
Convulsions
Unconsciousness
Muscular Twitching
Visual & Auditory Disturbances
Lightheadedness
Numbness of Tongue
Serum Concentration (µg/mL)
24
20
16
12
8
4
0
Relationship of signs and symptoms of lidocaine toxicity to serum concentration.21-23

## Slide 21
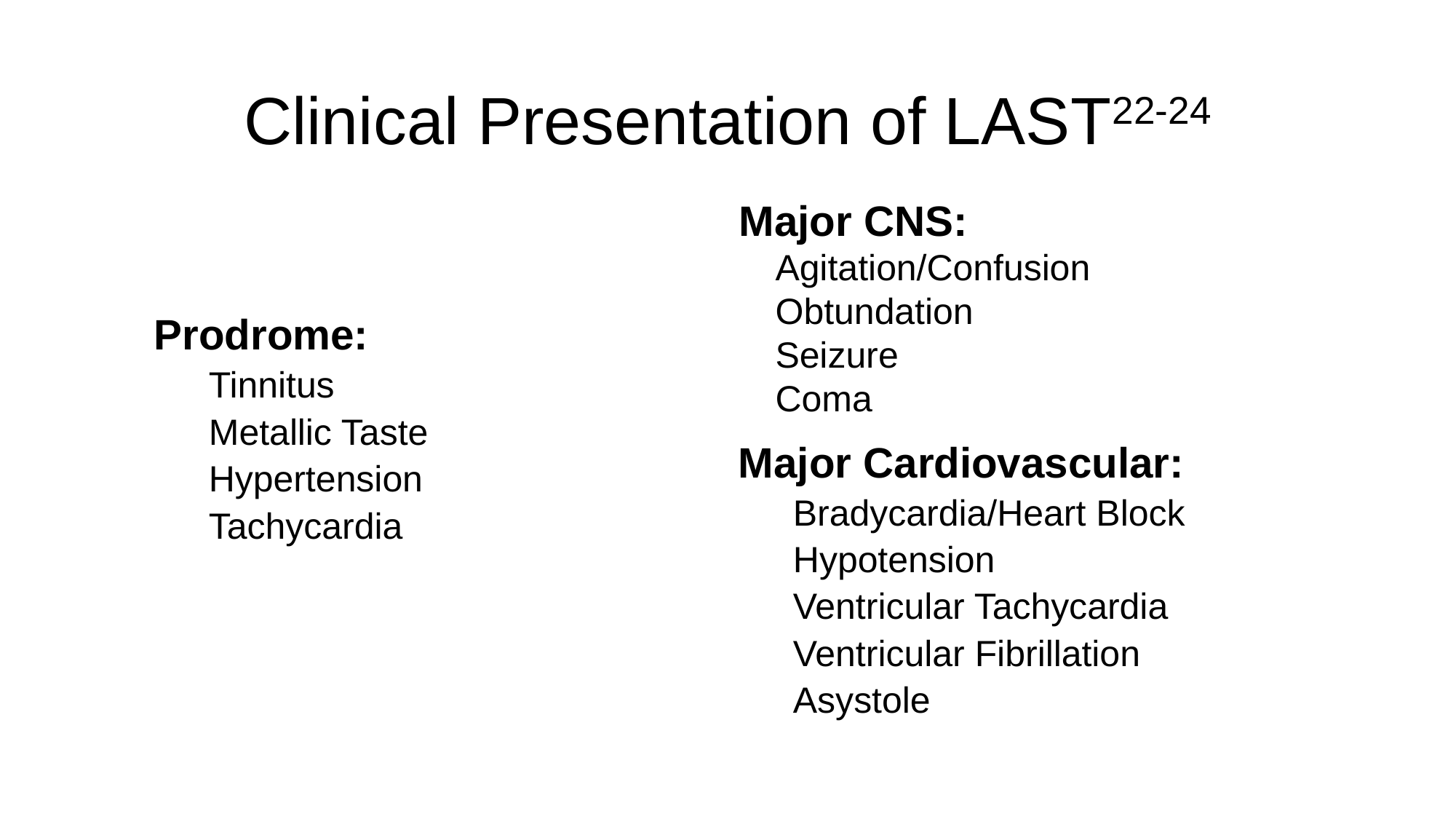

# Clinical Presentation of LAST22-24
Major CNS:​
 Agitation/Confusion ​
 Obtundation ​
 Seizure ​
 Coma
Prodrome:
Tinnitus
Metallic Taste
Hypertension
Tachycardia
Major Cardiovascular:
Bradycardia/Heart Block
Hypotension
Ventricular Tachycardia
Ventricular Fibrillation
Asystole

## Slide 22
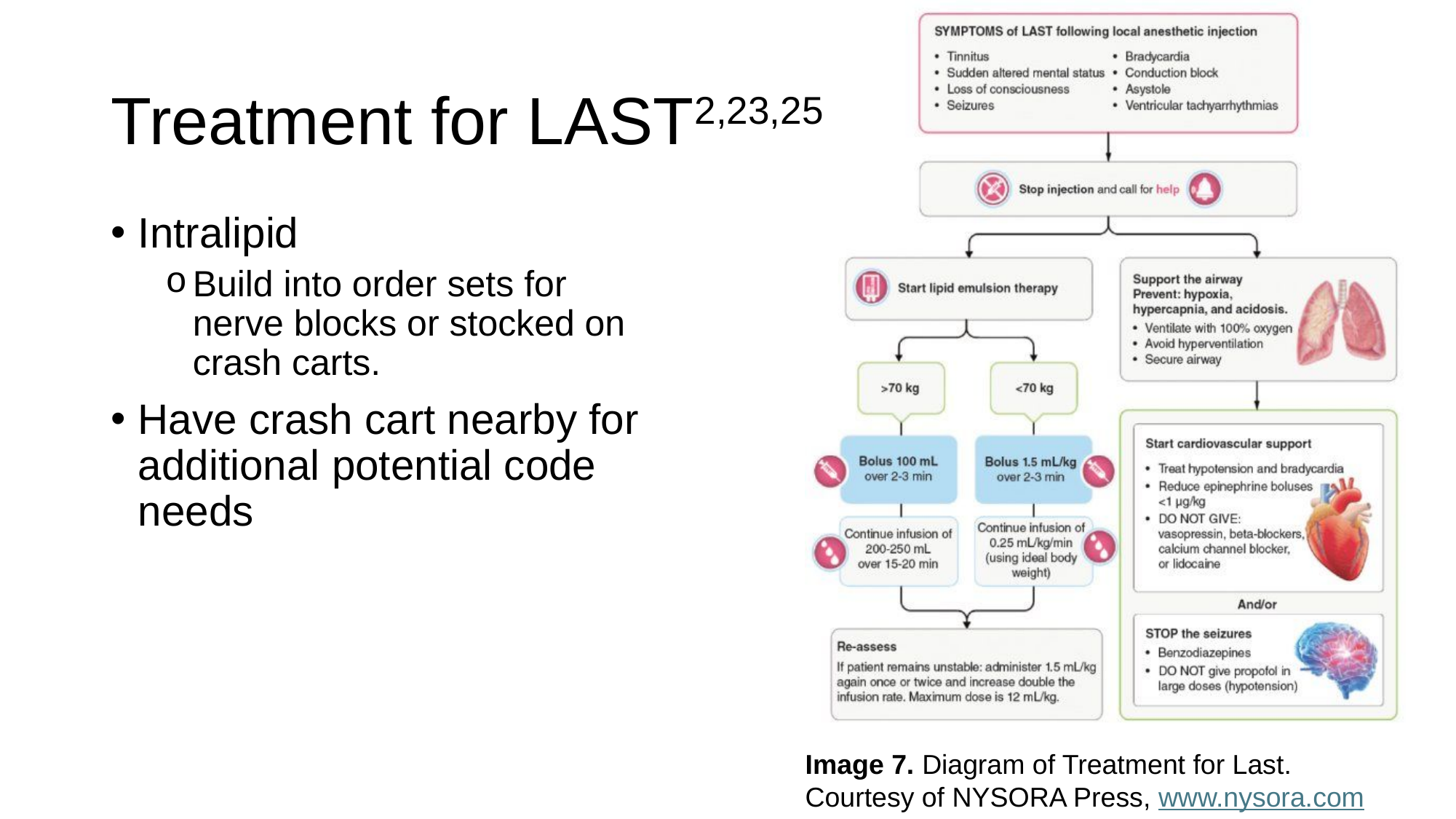

# Treatment for LAST2,23,25
Intralipid
Build into order sets for nerve blocks or stocked on crash carts.
Have crash cart nearby for additional potential code needs
Image 7. Diagram of Treatment for Last. Courtesy of NYSORA Press, www.nysora.com

## Slide 23
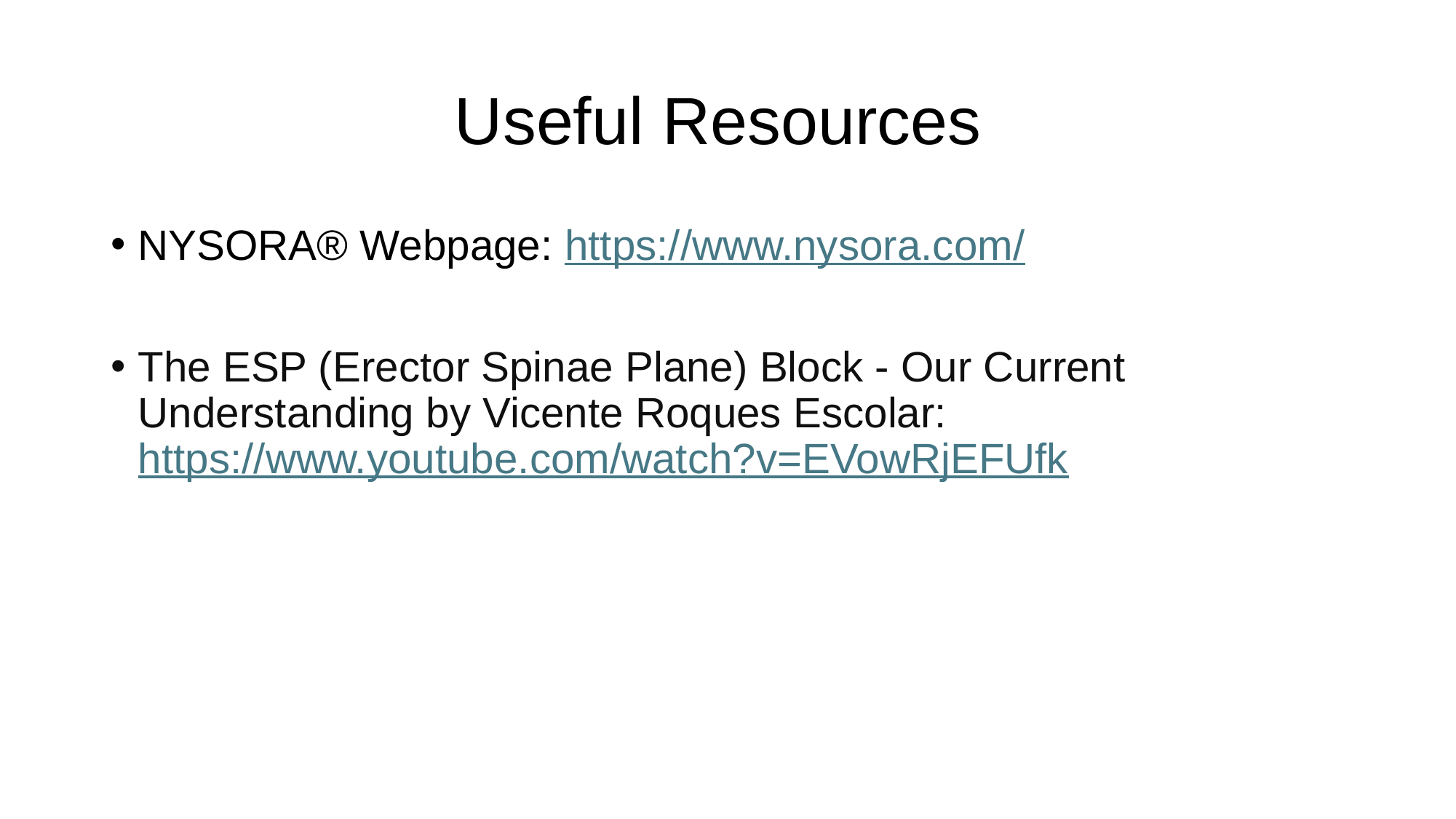

# Useful Resources
NYSORA® Webpage: https://www.nysora.com/
The ESP (Erector Spinae Plane) Block - Our Current Understanding by Vicente Roques Escolar: https://www.youtube.com/watch?v=EVowRjEFUfk

## Slide 24
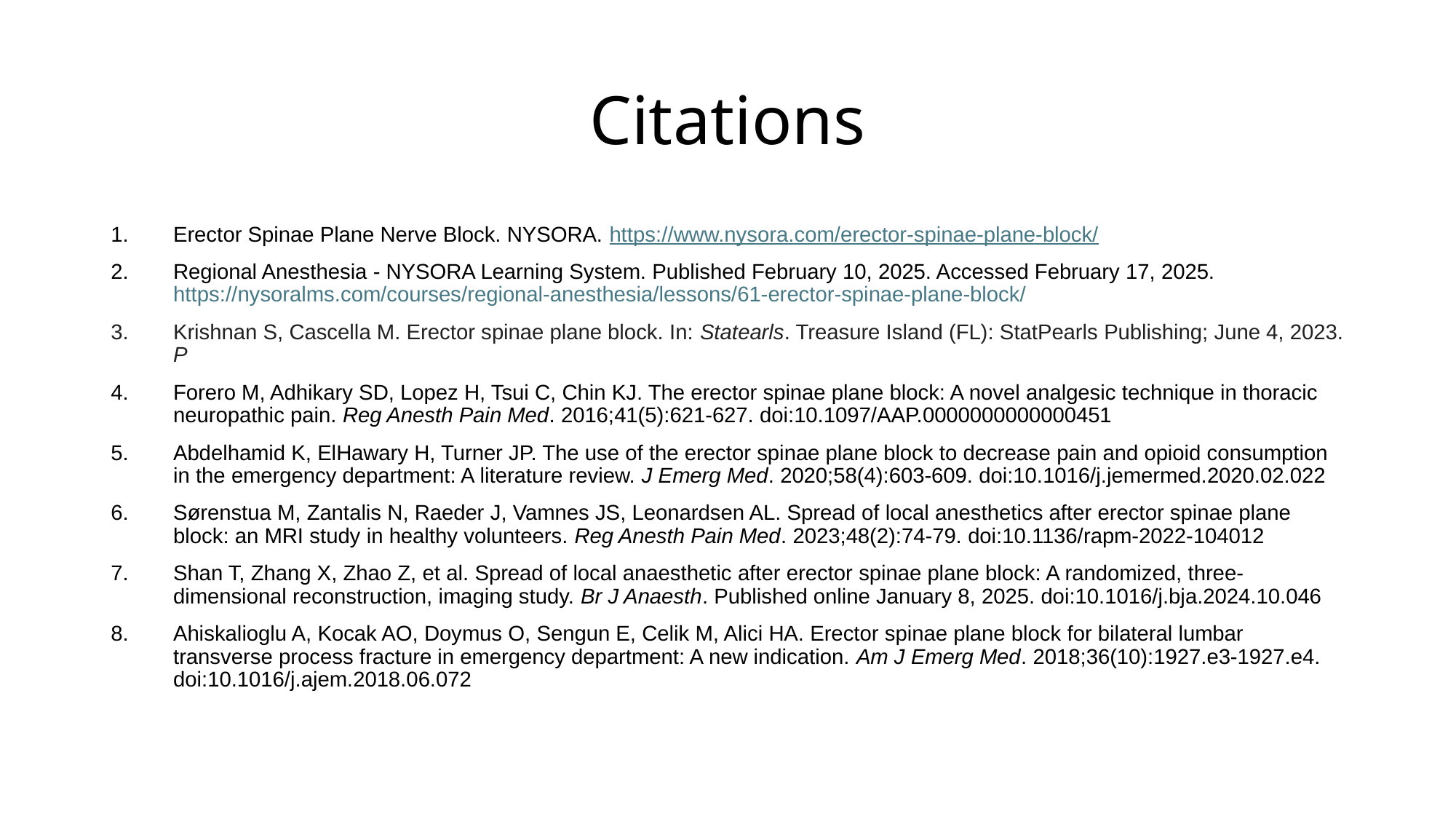

# Citations
Erector Spinae Plane Nerve Block. NYSORA. https://www.nysora.com/erector-spinae-plane-block/
Regional Anesthesia - NYSORA Learning System. Published February 10, 2025. Accessed February 17, 2025. https://nysoralms.com/courses/regional-anesthesia/lessons/61-erector-spinae-plane-block/
Krishnan S, Cascella M. Erector spinae plane block. In: Statearls. Treasure Island (FL): StatPearls Publishing; June 4, 2023. P
Forero M, Adhikary SD, Lopez H, Tsui C, Chin KJ. The erector spinae plane block: A novel analgesic technique in thoracic neuropathic pain. Reg Anesth Pain Med. 2016;41(5):621-627. doi:10.1097/AAP.0000000000000451
Abdelhamid K, ElHawary H, Turner JP. The use of the erector spinae plane block to decrease pain and opioid consumption in the emergency department: A literature review. J Emerg Med. 2020;58(4):603-609. doi:10.1016/j.jemermed.2020.02.022
Sørenstua M, Zantalis N, Raeder J, Vamnes JS, Leonardsen AL. Spread of local anesthetics after erector spinae plane block: an MRI study in healthy volunteers. Reg Anesth Pain Med. 2023;48(2):74-79. doi:10.1136/rapm-2022-104012
Shan T, Zhang X, Zhao Z, et al. Spread of local anaesthetic after erector spinae plane block: A randomized, three-dimensional reconstruction, imaging study. Br J Anaesth. Published online January 8, 2025. doi:10.1016/j.bja.2024.10.046
Ahiskalioglu A, Kocak AO, Doymus O, Sengun E, Celik M, Alici HA. Erector spinae plane block for bilateral lumbar transverse process fracture in emergency department: A new indication. Am J Emerg Med. 2018;36(10):1927.e3-1927.e4. doi:10.1016/j.ajem.2018.06.072

## Slide 25
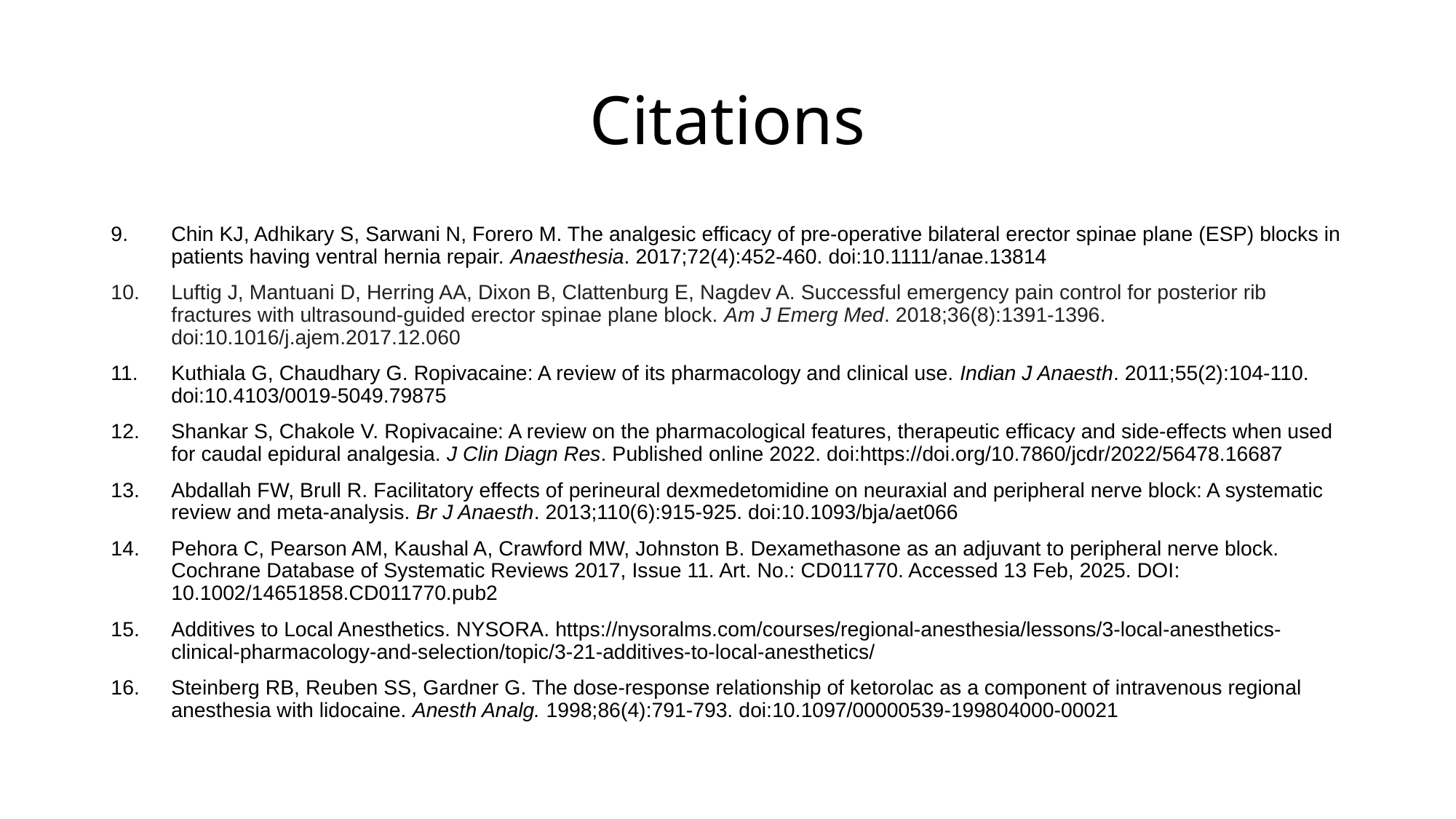

# Citations
Chin KJ, Adhikary S, Sarwani N, Forero M. The analgesic efficacy of pre-operative bilateral erector spinae plane (ESP) blocks in patients having ventral hernia repair. Anaesthesia. 2017;72(4):452-460. doi:10.1111/anae.13814
Luftig J, Mantuani D, Herring AA, Dixon B, Clattenburg E, Nagdev A. Successful emergency pain control for posterior rib fractures with ultrasound-guided erector spinae plane block. Am J Emerg Med. 2018;36(8):1391-1396. doi:10.1016/j.ajem.2017.12.060
Kuthiala G, Chaudhary G. Ropivacaine: A review of its pharmacology and clinical use. Indian J Anaesth. 2011;55(2):104-110. doi:10.4103/0019-5049.79875
Shankar S, Chakole V. Ropivacaine: A review on the pharmacological features, therapeutic efficacy and side-effects when used for caudal epidural analgesia. J Clin Diagn Res. Published online 2022. doi:https://doi.org/10.7860/jcdr/2022/56478.16687
Abdallah FW, Brull R. Facilitatory effects of perineural dexmedetomidine on neuraxial and peripheral nerve block: A systematic review and meta-analysis. Br J Anaesth. 2013;110(6):915-925. doi:10.1093/bja/aet066
Pehora C, Pearson AM, Kaushal A, Crawford MW, Johnston B. Dexamethasone as an adjuvant to peripheral nerve block. Cochrane Database of Systematic Reviews 2017, Issue 11. Art. No.: CD011770. Accessed 13 Feb, 2025. DOI: 10.1002/14651858.CD011770.pub2
Additives to Local Anesthetics. NYSORA. https://nysoralms.com/courses/regional-anesthesia/lessons/3-local-anesthetics-clinical-pharmacology-and-selection/topic/3-21-additives-to-local-anesthetics/
Steinberg RB, Reuben SS, Gardner G. The dose-response relationship of ketorolac as a component of intravenous regional anesthesia with lidocaine. Anesth Analg. 1998;86(4):791-793. doi:10.1097/00000539-199804000-00021

## Slide 26
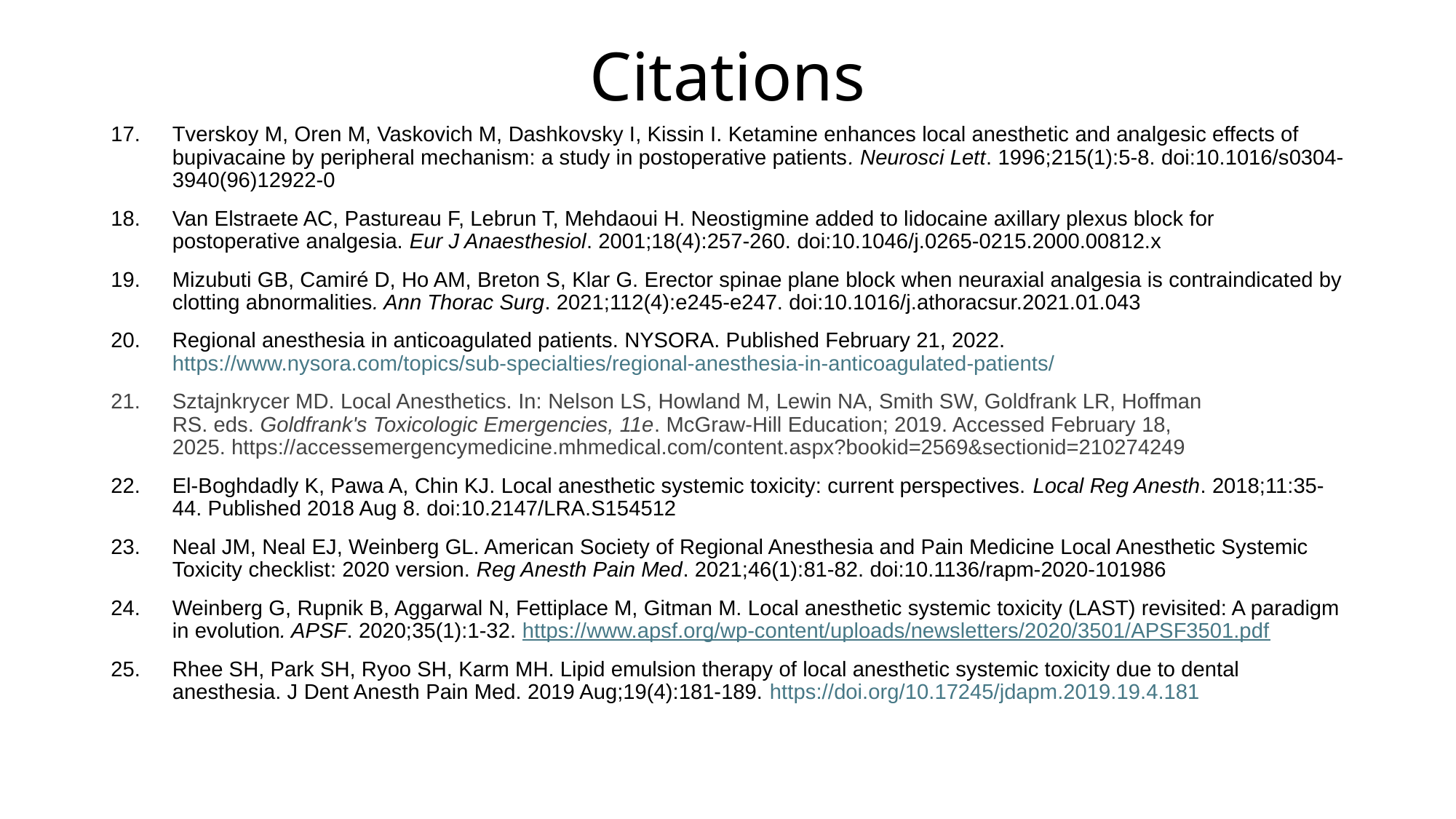

# Citations
Tverskoy M, Oren M, Vaskovich M, Dashkovsky I, Kissin I. Ketamine enhances local anesthetic and analgesic effects of bupivacaine by peripheral mechanism: a study in postoperative patients. Neurosci Lett. 1996;215(1):5-8. doi:10.1016/s0304-3940(96)12922-0
Van Elstraete AC, Pastureau F, Lebrun T, Mehdaoui H. Neostigmine added to lidocaine axillary plexus block for postoperative analgesia. Eur J Anaesthesiol. 2001;18(4):257-260. doi:10.1046/j.0265-0215.2000.00812.x
Mizubuti GB, Camiré D, Ho AM, Breton S, Klar G. Erector spinae plane block when neuraxial analgesia is contraindicated by clotting abnormalities. Ann Thorac Surg. 2021;112(4):e245-e247. doi:10.1016/j.athoracsur.2021.01.043
Regional anesthesia in anticoagulated patients. NYSORA. Published February 21, 2022. https://www.nysora.com/topics/sub-specialties/regional-anesthesia-in-anticoagulated-patients/ ‌
Sztajnkrycer MD. Local Anesthetics. In: Nelson LS, Howland M, Lewin NA, Smith SW, Goldfrank LR, Hoffman RS. eds. Goldfrank's Toxicologic Emergencies, 11e. McGraw-Hill Education; 2019. Accessed February 18, 2025. https://accessemergencymedicine.mhmedical.com/content.aspx?bookid=2569&sectionid=210274249
El-Boghdadly K, Pawa A, Chin KJ. Local anesthetic systemic toxicity: current perspectives. Local Reg Anesth. 2018;11:35-44. Published 2018 Aug 8. doi:10.2147/LRA.S154512
Neal JM, Neal EJ, Weinberg GL. American Society of Regional Anesthesia and Pain Medicine Local Anesthetic Systemic Toxicity checklist: 2020 version. Reg Anesth Pain Med. 2021;46(1):81-82. doi:10.1136/rapm-2020-101986
Weinberg G, Rupnik B, Aggarwal N, Fettiplace M, Gitman M. Local anesthetic systemic toxicity (LAST) revisited: A paradigm in evolution. APSF. 2020;35(1):1-32. https://www.apsf.org/wp-content/uploads/newsletters/2020/3501/APSF3501.pdf
Rhee SH, Park SH, Ryoo SH, Karm MH. Lipid emulsion therapy of local anesthetic systemic toxicity due to dental anesthesia. J Dent Anesth Pain Med. 2019 Aug;19(4):181-189. https://doi.org/10.17245/jdapm.2019.19.4.181
